# Data-Driven Thresholding Statistically Biases ATN Profiling across Cohort Datasets

**DOI:** 10.14283/jpad.2023.100

**Published:** 2023-09-06

**Authors:** Y. Salimi, D. Domingo-Fernández, M. Hofmann-Apitius, C. Birkenbihl, the Alzheimer’s Disease Neuroimaging Initiative, the Japanese Alzheimer’s Disease Neuroimaging Initiative, the Alzheimer’s Disease Repository Without Borders Investigators, the European Prevention of Alzheimer’s Disease (EPAD) Consortium

**Affiliations:** 1 Department of Bioinformatics, Fraunhofer Institute for Algorithms and Scientific Computing (SCAI), Sankt Augustin, 53757 Germany; 2 Bonn-Aachen International Center for IT, Rheinische Friedrich-Wilhelms-Universität Bonn, Bonn, 53115 Germany; 3 Schloß Birlinghoven, Sankt Augustin, 53757 Germany

**Keywords:** ATN framework, biomarker profiles, CSF thresholds, Alzheimer’s disease, cohort studies

## Abstract

**Background:**

While the amyloid/tau/neurodegeneration (ATN) framework has found wide application in Alzheimer’s disease research, it is unclear if thresholds obtained using distinct thresholding methods are concordant within the same dataset and interchangeable across cohorts.

**Objectives:**

To investigate the robustness of data-driven thresholding methods and ATN profiling across cohort datasets.

**Design and Setting:**

We evaluated the impact of thresholding methods on ATN profiles by applying five commonly-used methodologies across cohort datasets. We assessed the generalizability of disease patterns discovered within ATN profiles by clustering individuals from different cohorts who were assigned to the same ATN profile.

**Participants and Measurements:**

Participants with available CSF amyloid-β 1–42, phosphorylated tau, and total tau measurements were included from eleven AD cohort studies.

**Results:**

We observed high variability among obtained ATN thresholds, both across methods and datasets that impacted the resulting profile assignments of participants significantly. Clustering participants from different cohorts within the same ATN category indicated that identified disease patterns were comparable across most cohorts and biases introduced through distinct thresholding and data representations remained insignificant in most ATN profiles.

**Conlusion:**

Thresholding method selection is a decision of statistical relevance that will inevitably bias the resulting profiling and affect its sensitivity and specificity. Thresholds are likely not directly interchangeable between independent cohorts. To apply the ATN framework as an actionable and robust profiling scheme, a comprehensive understanding of the impact of used thresholding methods, their statistical implications, and a validation of results is crucial.

**Electronic Supplementary Material:**

Supplementary material is available in the online version of this article at 10.14283/jpad.2023.100.

## Introduction

Alzheimer’s disease (AD) is a progressive condition in which symptoms manifest years after the initial onset of the disease (1). Over the past decades, AD diagnosis relied predominantly on cognitive assessments, and individuals were commonly diagnosed into i) cognitively unimpaired (CU), ii) mild cognitive impairment (MCI), or iii) AD. Patients within the cognitively impaired groups, however, exhibit a large degree of heterogeneity with respect to symptoms (2), disease severity, and progression (3). One proposed reason for this was that a clinical definition of AD ignores the disease-underlying biological condition of patients such as their state of amyloid burden and neurodegeneration (4).

Motivated by these concerns, the β-amyloid deposition (A), pathologic tau (T), and neurodegeneration (N) framework was proposed as a potentially unbiased approach for categorizing AD patients according to their biological condition rather than cognitive function (1). The ATN framework refers to a specific set of biomarkers to measure whether any of the three factors appear abnormal. Based on this categorization scheme, participants are then assigned an abnormal (+), or normal (−) state for each factor, resulting in eight possible biomarker profiles (e.g., A+T+N+, A+T+N, etc.). For such a categorization of biomarker states, biomarker-specific thresholds need to be defined. To this end, predominantly data-driven methods have been used including clustering approaches such as K-means and Gaussian mixture models (GMM), as well as, for example, placing a threshold at a specific quantile of the empirical data distribution.

While over a hundred published studies have employed the ATN framework to profile individuals, the majority of them applied different thresholds, defined using select data-driven techniques, relying on single cohort datasets. Comparing such thresholds across different assays used to measure the biomarkers remains difficult due to a lack of assay standardization (5). However, even if such thresholds were derived from data measured via the standardized assays, they are bound to be impacted by distribution shifts arising from the particulars of patient recruitment, data collection, and processing that are inherent to cohort datasets (6). Therefore, to ensure that ATN-based results do generalize across AD populations, it is essential to evaluate whether disparate thresholding methods result in different thresholds due to data characteristics and how far such differences could bias the resulting ATN profiling. Lastly, it remains unclear whether participants of one cohort study would indeed be comparable to another cohort’s participants who were both assigned to the same ATN profile based on different, purely data-determined thresholds.

Previously, studies were conducted investigating the selection of ATN biomarkers, the method for defining thresholds (7, 8), and whether thresholds were interchangeable across two cohorts (9). One study found that cerebrospinal fluid (CSF) thresholds achieved from the Alzheimer’s Disease Neuroimaging Initiative (ADNI) could be applied to the BioFINDER cohort when adjusting for preanalytical differences (9). Another recent study inspected potential differences in ATN profiles associated with the choice of biomarkers as well as the method for dichotomization, again using ADNI-derived thresholds on BioFINDER (7). They found few differences among thresholds obtained using different methodologies, except for CSF amyloid-β 1–42 (Aβ1-42) thresholds. A further study leveraged the European Prevention of Alzheimer’s Dementia (EPAD) dataset and reported that GMM thresholds aligned with literature-reported values (8). However, such previous studies focused on a limited number of cohorts, and the question of generalizability, meaning whether disease patterns exhibited by participants assigned to the same ATN profile are comparable across cohorts, was not investigated. In order for the ATN framework to present an actionable, universal, and unbiased profiling scheme based on participants’ biological conditions, a comprehensive understanding of applied thresholding methods, their impact, and the generalizability of achieved results is crucial.

In this work, we identified a set of eleven AD cohorts that contained the CSF biomarkers recommended for applying the ATN framework (i.e., Aβl-42, phosphorylated tau 181 (pTau), and total tau (tTau)) (1). Subsequently, we used five well-established methods to define thresholds for each biomarker in each cohort to categorize its participants according to the ATN framework. Following this, we analyzed deviations among the thresholds defined by each method and investigated the impact of such deviations on the underlying profiling. Lastly, we evaluated whether individuals assigned to the same ATN profiles exhibited similar disease patterns across cohorts, despite their different biomarker thresholds.

## Methods

### Investigated cohort studies

Using the ADataViewer (6), we identified eleven cohort studies that measured the CSF biomarkers necessary for ATN profiling (Table 1) (1).

**Table 1:** Investigated cohort studies and their respectively employed CSF assays

**Cohort**	**Consortium name**	**CSF assay**
ADNI	The Alzheimer’s Disease Neuroimaging Initiative (11)	Fully automated Roche Elecsys immunoassay
EPAD	European Prevention of Alzheimer’s Dementia V.IMI (12)	
AIBL	The Australian Imaging, Biomarker & Lifestyle Flagship Study of Ageing (13)	INNOTEST® kit assay (Innogenetics, Ghent, Belgium), Enzyme-linked immunosorbent assay (ELISA)
ARWIBO	Alzheimer’s Disease Repository Without Borders (14)	
EDSD	The European DTI Study on Dementia (15)	
PREVENT-AD	Pre-symptomatic Evaluation of Experimental or Novel Treatments for Alzheimer’s Disease (16)	
PharmaCog	Prediction of Cognitive Properties of New Drug Candidates for Neurodegenerative Diseases in Early Clinical Development (17)	
NACC	The National Alzheimer’s Coordinating Center (18)	INNOTEST® kit assay (Innogenetics, Ghent, Belgium), Enzyme-linked immunosorbent assay (ELISA) Multiplex xMAP Luminex platform (LuminexCorp., Austin, TX, USA) with Innogenetics (INNO-BIA AlzBio3, Ghent, Belgium) immunoassay
EMIF	European Medical Information Framework (19)	
DOD-ADNI	Effects of TBI & PTSD on Alzheimer’s Disease in Vietnam Vets (20)	Multiplex xMAP Luminex platform (LuminexCorp., Austin, TX, USA) with Innogenetics (INNO-BIA AlzBio3, Ghent, Belgium) immunoassay
JADNI	Japanese Alzheimer’s Disease Neuroimaging Initiative (21)	

Cohorts are grouped together if they employed the same assay(s). Note that EMIF and NACC used two distinct assays.

To maximize the number of analyzable participants, we focused on measurements taken at each cohort study’s respective baseline. The number of participants per cohort as well as summary statistics of demographic variables are shown in Table S1. NACC and EMIF contained CSF measurements acquired using different assays, therefore, we divided their participants into separate groups based on the assay type (Table S2).

### CSF biomarkers

We restricted our analysis to CSF biomarkers as they were widely available across cohorts and, according to the ATN framework, CSF biomarkers alone are sufficient for ATN profiling (1). The selected CSF biomarkers were Aβl-42, pTau181 (pTau), and tTau indicating A, T, and N, respectively (i.e., N+ indicates abnormal tTau levels while N- indicates that tTau were normal). Aβ1-42 was considered abnormal if the measurement was below the threshold, while pTau and tTau were determined abnormal if the measurement was higher than a given value (4, 10). The assays used in each cohort are presented in Table 1. Measurement values below or above the assay-specific technical limit were (Table S3). The distribution of each CSF biomarker for cohorts with the same assay is shown in Fig. S1.

### Thresholding methods

We reviewed 116 publications using the ATN framework by querying PubMed with [ATN + “Alzheimer’s disease”] with the goal of identifying the most commonly used methods for obtaining biomarker thresholds (Table S4). Our survey revealed five data-driven methods as frequently used: 1) GMM, 2) K-means clustering, 3) tertile analysis, 4) receiver operating characteristic (ROC) analysis, and 5) mean ±2 standard deviation (SD). We applied these methods to each cohort individually to define a threshold. To gain robust threshold estimates, we calculated the 95% Confidence Intervals (CIs) for each threshold based on 1,000 bootstrap samples. We want to emphasize that we deliberately followed the exact procedures reported in the literature to highlight the impact of commonly applied methods.

For the two clustering methods (i.e., GMM and K-means), a clustering solution with two clusters was sought (representing normal and abnormal biomarker values, respectively). When employing GMM, the value at which the two identified Gaussians overlapped was selected as a threshold. For K-means, the average distance between the two clusters’ centroids represented the threshold. In both of these approaches, the clinical diagnoses of participants were not considered and, like in the majority of publications utilizing this approach, participant age was added as an additional dimension to the clustering. In contrast, ROC analysis, tertile, and mean ±2 SD required the diagnoses of participants to define thresholds. When using ROC analysis, the threshold that yielded the highest Youden’s index and thus indicated the best separation of AD and CU participants was chosen (22, 23). The tertile and mean ±2 SD methods rely on the biomarker distributions of CU participants to define thresholds (7, 24). Here, the tertile method considered values to be abnormal if they fell within the highest tertile of the distribution for tTau and pTau, and within the lowest tertile for Aβ1-42. Correspondingly, the mean ±2 SD method defined values as abnormal if they were at least 2 standard deviations greater than the mean for tTau and pTau, and lower for Aβ1-42. In some cohorts, the diagnosis-reliant methods could not be applied due to an insufficient number of participants within either of the required diagnostic groups (i.e., less than 10 CU or AD patients).

### Evaluating the concordance of ATN profiles across cohorts

To investigate if participants of different cohorts assigned to the same ATN profile are comparable and whether profilings based on different, study-specific thresholds would exhibit similar disease patterns, we performed hierarchical clustering on participants from all cohorts within their respective ATN profiles. Limited comparability between participants would be reflected by a clustering that is primarily driven by dataset membership, with participants from the same cohort tending to cluster together (i.e., a high correlation between cluster labels and cohort membership). Conversely, an evenly spread-out distribution of participants across clusters irrespective of their cohort membership indicates that similar disease patterns can be found across cohorts despite differences in ATN thresholds.

Seven cohorts were included in this analysis (ADNI, ARWIBO, DOD-ADNI, EDSD, JADNI, NACC, and PharmaCog), as finding a sufficient variable overlap between more cohorts proved infeasible. The clustering was based on 106 shared variables, 104 of which were MRI-measured brain region volumes with the remaining two being the Apolipoprotein E ε4 (APOE ε4) status, and Mini-Mental State Examination (MMSE) (Table S5). In order to adjust for the presence of potential batch effects in MRI variables, as caused, for example, by differences across scanners used across studies, we applied the pyComBat batch-correction (25). Clustering distances were calculated using Ward-linkage. The analysis was performed for K-Means and GMM-derived thresholds for participant profiling, respectively. ATN profiles with less than 20 assigned participants were excluded from this analysis. To measure the association between dataset membership and cluster labels, we calculated Cramer’s V with bias correction (26) and assessed statistical significance using a chi^2^-test assuming a confidence level of 95%.

We conducted the hierarchical clustering using two procedures for determining the sought-after number of clusters per ATN profile. First, we optimized the number of clusters by calculating the silhouette index across a range of possible clusterings and selected the number of clusters that maximized this index. In a second, more naive attempt, we assigned the number of clusters equal to the number of cohorts considered for each profile.

To provide an additional perspective, we applied the UMAP algorithm (27) to all participants of the previously mentioned seven cohorts who were assigned to each ATN profile. UMAP projects a dataset into a lower dimensional space (here, two dimensions) while trying to preserve the global structure of the data. For this, we used the same 106 variables as in the clustering (Table S5). Again, ATN profiles with less than 20 participants were excluded. The resulting lower-dimensional visualization provides a notion of whether participants assigned to the same ATN profile could be easily separated by cohort membership.

## Results

### Variation of data-driven thresholds obtained using different methods

When comparing the thresholds yielded by applying our five selected thresholding approaches to each of the eleven cohorts, we observed considerable differences (Table 2). For example, the thresholds obtained for Aβ1-42 in ADNI ranged from 320.8 (mean ±2 SD) to 1,136.4 (GMM). In the EMIF_ELISA subgroup (i.e., EMIF participants with CSF values measured using ELISA), the thresholds for tTau varied from 269.4 (tertile) to 669.4 (mean ±2 SD). Generally, the largest difference across estimated threshold values was found for Aβ1-42 with an average deviation of 66%, calculated with respect to each cohort’s largest observed Aβ1-42 threshold (Table S8). For pTau and tTau, we discovered average deviations of 45% and 49%, respectively.

**Table 2:** Thresholds obtained for each CSF biomarker using different data-driven methods

**Cohort**	**Method**														
	**GMM**			**K-means**			**Tertile**			**ROC**			**Mean ±2 SD**		
	**Aβ1-42**	**pTau**	**tTau**	**Aβ1-42**	**pTau**	**tTau**	**Aβ1-42**	**pTau**	**tTau**	**Aβ1-42**	**pTau**	**tTau**	**Aβ1-42**	**pTau**	**tTau**
ADNI	1136.4	35.3	364.9	1095	27.5	285.8	951.4	23.7	259.1	888.1	24.3	265.9	320.8	40.2	418.4
EPAD	1385	29.2	312.2	1180.5	19.6	224.8	987.1	20.3	238.1	731.5	19.1	213.8	366.8	40.6	422.1
AIBL	714.4	92.6	591.2	689.9	77.7	635.3	-	-	-	-	-	-	-	-	-
ARWIBO	580.7	120	524.6	511.1	72	559.8	-	-	-	-	-	-	-	-	-
EDSD	737.3	112.8	524.7	790.8	82.8	577.7	-	-	-	-	-	-	-	-	-
PharmaCog	766.3	93.1	671.6	773.6	66.2	465.8	-	-	-	-	-	-	-	-	-
PREVENT-AD	1015.8	68	424.4	1150.4	50.3	301.6	1104.4	53.1	304.2	-	-	-	588.4	89.6	598.1
NACC_ELISA	596.2	80.8	537.4	589.1	61.1	497.4	629	48	376.6	474	64	468	291.5	88	728.8
EMIF_ELISA	743.6	81.4	523.8	723.7	65	435.1	538.2	52.1	269.4	560	59	355.4	173.1	95.1	669.4
NACC_xMAP	298.6	58.4	94	270.3	40.2	70	244.8	36.3	53.8	226.4	41.2	56.7	21.8	69.3	99.7
EMIF_xMAP	400.4	51.7	204.2	356.4	37.6	129.9	450.2	30.8	83.2	352.2	28.7	100.1	239.5	55.2	123.4
DOD-ADNI	1276	26.5	306.1	1231.9	22.5	250.2	927	19.8	229	-	-	-	282.4	34.3	369.9
JADNI	416.4	61.3	133.8	414.3	62	145.8	392	38.9	72.2	333.9	44.9	86.2	186.9	66.2	128.2

′-′: Method application infeasible due to an insufficient number of CU or AD patients in the respective cohort. The NACC and EMIF cohorts were divided into separate groups based on the CSF assay used for measuring the CSF biomarkers within each cohort. All values are given as pg/mL. In order for Aβ1-42 measurements to be considered abnormal, the measurement needs to place below the threshold, while for Tau biomarkers an abnormal value exceeds the threshold.

To further evaluate the robustness of the threshold estimates, we bootstrapped each respective cohort’s participants and calculated 95 % CIs for the estimate (Table S6). Across all cohorts and thresholding methods, the uncertainty of estimated values remained rather small in the majority of cases (below 1% relative difference with respect to the upper CI bound) with a maximum relative difference of 8.8% for the pTau threshold of ARWIBO estimated using GMM (95% CI [137-150]; Table S7). Surprisingly, only 40 of the 153 thresholds obtained on the full cohort datasets placed within their corresponding CI, however, we found that most deviations were numerically small and clinically neglectable (e.g., ADNI tTau threshold estimated using ROC of 266 versus a 95% CI of [268, 270]). Exceptionally large deviations were found, for example, for the GMM-derived Aβ1-42 threshold in PREVENT_AD (full cohort point estimate: 1,016; 95% CI: [1,173-1,232]), or the K-means-determined tTau threshold in AIBL (full cohort: 635; 95% CI: [519-533]).

**Figure 1:**
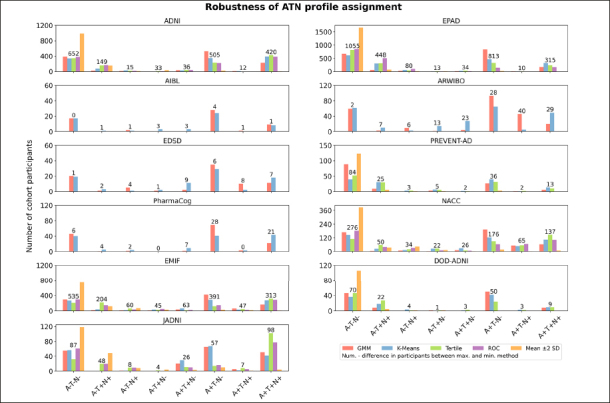
Proportion of participants categorized to each ATN profile by the different thresholding techniques across cohorts

In general, we found that the most conservative profiling thresholds (i.e., yielding numerically more extreme thresholds that make it less likely for biomarker measurements to be considered abnormal) were obtained using the mean ±2 SD method (Table 2; Figure 1), while the clustering-based approaches (K-means and GMM) and ROC analysis often estimated thresholds that were less conservative. The tertile method commonly was the least conservative.

The y-axis shows the proportion of participants assigned to each of the eight biomarker profiles in each cohort. The absolute number of patients assigned for each category is displayed in Supplementary Tables S11–S15.

### Evaluating the robustness of data-driven thresholds across cohorts

We investigated whether thresholds were similar across cohorts using the same assay and found that the majority of them varied substantially (Table 2). Among the seven cohorts using the INNOTEST® assays, the average across-cohort difference of Aβ1-42, pTau, and tTau thresholds respectively amounted to 47%, 21%, and 32% of the highest value per biomarker (Table S9). Here, especially the Aβ1-42 values of PREVENT-AD showed substantial deviations from values gained in other cohorts. Among the four cohorts employing a multiplex xMAP assay, we found average differences of 71%, 51%, and 67% for Aβ1-42, pTau, and tTau, respectively. Only the thresholds of cohorts using the Roche Elecsys immunoassay (ADNI and EPAD) were relatively similar with an average difference of 12%, 17%, and 13% of the corresponding highest value of Aβ1-42, pTau, and tTau, respectively.

When investigating which of the methods obtained the most consistent estimates across cohorts and biomarkers, we found that the mean ±2 SD method was most consistent for the Roche Elecsys immunoassay datasets (average difference across cohorts and biomarkers of 5%) (Table S10). Further, thresholds estimated using ROC analysis showed the lowest deviation for the INNOTEST® assay, as well as for the cohorts using a multiplex xMAP assay-employing cohorts with a respective average difference of 16% and 38% across cohorts and biomarkers.

### Impact of thresholding method selection on ATN profiling

Per cohort, we investigated how the assignment of its participants to ATN profiles changed when distinct thresholding methodologies were applied (Number of samples per cohort, method, and ATN profile are provided in Tables S11–S15). Unsurprisingly, given the observed magnitude of threshold differences (Table 2), the method selection substantially influenced the achieved profiling, as vast differences in sample size per ATN profile were identified (Figure 1). In alignment with the previously presented results, depending on how conservative the methods were, fewer or more participants were assigned to ATN profiles that involved abnormal biomarker measurements. Here, the largest contrasts in participant assignments between methods commonly involved the mean ±2 SD approach, which assigned most participants to the A-T-N- profile. GMM and K-means often returned quite similar participant counts, while the tertile method assigned relatively more individuals to the A+T+N+ profile. Large deviations between the distinctly thresholded assignments were, for example, observed in EPAD, where the difference between the highest and lowest participant counts amounted to 1,055 individuals for A−T−N−, 448 for A−T+N+, 813 for A+T−N−, and 315 for A+T+N+ (total number of EPAD participants: 1,776). For some biomarker profiles and cohorts, an interpretation of the observed participant counts remained difficult due to limited sample sizes (e.g., AIBL).

### Generalizability of ATN profile-specific disease patterns across cohorts

To evaluate if ATN-based results can be generalized, we conducted a hierarchical clustering of participants from seven cohorts within each ATN profile based on MRI measurements, APOE ε4 status, and MMSE. The ATN profiles were defined using K-means and GMM-obtained thresholds, respectively. MRI measurements were adjusted for batch effects as, for example, introduced by using different scanners across cohorts. Finally, the correlation between cluster assignments and cohort membership was assessed to evaluate if individuals cluster together based on disease patterns or shared cohort membership.

When determining the optimal number of clusters for partitioning participants, we found that already two or three clusters provided the best clustering solution for each ATN profile (Figure S2). We identified no significant associations between cluster labels and cohort membership in ATN profiles thresholded using K-means (p>0.05; Table 3), indicating that the clustering was likely not dominated by individuals’ cohort origin. Only when applying GMM for thresholding, significant and strong associations were discovered in the A+T+N− (Cramer’s V=0.41, p<0.01) and A+T−N+ (Cramer’s V=0.3, p<0.04) profiles.

**Table 3:** The Cramer’s V and the p-value for each clustering of participants in each ATN profile using certain data-driven thresholds

**Method**		**Biomarker Profile**						
		**A-T-N-**	**A-T+N+**	**A-T-N+**	**A+T+N-**	**A+T-N-**	**A+T-N+**	**A+T+N+**
K-means	Cramer’s V	0.15	0.33	0.33	0.21	0.14	0.1	0.2
	Chi^2^ p-value	0.94	0.61	0.35	0.65	0.61	0.75	0.56
	Total Participants	450	78	30	75	434	35	364
GMM	Cramer’s V	0.17	0.33	0.19	0.41	0.13	0.3	0.25
	Chi^2^ p-value	0.3	0.82	0.61	0.01	0.37	0.04	0.07
	Total Participants	500	25	26	40	588	67	232

Note: The ATN profile with less than 20 participants was excluded (i.e., A-T+N-).

In a second variation of the analysis, we set the number of clusters equal to the number of cohorts per ATN profile. We observed significant associations between cohort membership and cluster assignment for GMM-defined A+T+N− (Cramer’s V=0.36, p<0.01) as well as A+T+N+ (Cramer’s V=0.16, p<0.02) profiles, respectively (Table S18).

**Figure 2:**
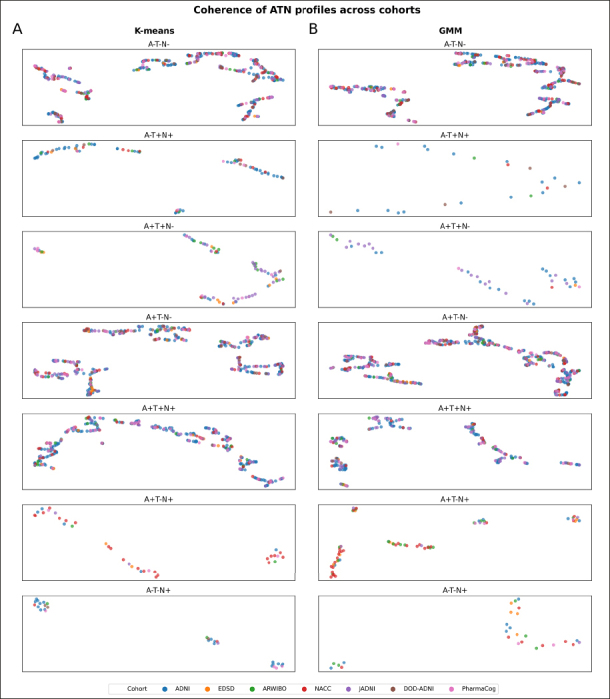
Identification of potential participants’ subgroups categorized in each ATN profile among cohorts using UMAP

To provide a visual intuition about how similar individuals of the same ATN profile were across cohorts, we generated a UMAP visualization based on the same cohorts and variables used in the previously mentioned clustering approach (Figure 2; the total number of participants per cohort that could be included in this analysis is presented in Tables S16–S17). While interpreting absolute distances in a UMAP is futile, the grouping of individuals from different cohorts and the relative distances between participants of the same cohort compared to the distances observed between participants from distinct cohorts provide an indication of their similarity.

A) ATN profiles achieved using K-means thresholds. B) ATN profiles achieved using GMM thresholds. Note: Missing profiles were removed due to the lack of participants (number of participants below 20). No axis labeling is provided as they are not directly interpretable. The total number of participants in each ATN profile is presented in supplementary Tables S16–S17.

In ATN profiles defined using both K-means (Figure 2A) and GMM (Figure 2B) respectively, we observed individuals from multiple cohorts in all visible aggregations. Simultaneously, members from each cohort distributed widely across the UMAP space and were often positioned closer to other datasets’ participants than their peers. Especially in the more populated biomarker profiles (A−T−N−, A+T−N−, and A+T+N+), we observed no clear separation of participants by dataset. Thorough interpretation of sparser profiles, however, remained difficult due to unequal and sometimes low sample sizes of cohorts. Conclusively, both the clustering approach and UMAP projection seemed largely not governed by cohort membership, but by actual disease signals.

## Discussion

In this work, we investigated the robustness and generalizability of the ATN framework across eleven AD cohort datasets and five commonly used data-driven approaches for defining biomarker thresholds. When comparing thresholds yielded by distinct methods for the same biomarker, we observed substantial variation that showcased the methods’ contrasting statistical properties and the impact that method selection has on ATN profiling. Even when CSF biomarkers were measured using the same assay, applying the same thresholding method led to estimates that often deviated substantially across cohorts. This indicates that thresholds are most likely not interchangeable across cohorts without disrupting the ATN profile assignments of individuals. Further, it is not certain that profiling of distinct cohort datasets necessarily assigns individuals to the same ATN profile who exhibit comparable disease patterns. However, clustering participants of seven cohorts within each ATN profile seldom revealed cluster separation by dataset membership, indicating that participants with similar disease signals could be identified from individually profiled cohorts. This showed that patterns discovered in independently ATN profiled cohorts could still be comparable even though numerical differences exist in the respectively applied thresholds.

### Determined thresholds are dataset and method-specific

Determining thresholds for CSF Aβ1-42, pTau, and tTau using five established thresholding methods across datasets revealed large variations depending on which method and dataset were used. We speculate that these differences find their origin in the recruitment criteria of cohorts that define the population from which their participants were enrolled (6), as well as variation in biomarker assessment (28, 29). Additionally, the identified differences could be promoted by assumptions that thresholding methods make on the data from which thresholds are estimated. For instance, when applying methods that rely on the clinical diagnosis of participants, thresholds could be distinctly influenced by discrepant diagnostic criteria or differences in the average disease stage of participants. The AD diagnosis in PharmaCog, for example, was made based on amnestic MCI with low Aβ1-42 CSF levels (17), while AIBL participants were diagnosed using the NINCDS-ADRDA criteria (13).

Whether thresholds are interchangeable between cohorts depends strongly on the data they were estimated from, the applied thresholding method, and most importantly whether employed assays were standardized. As current assays are research-grade, their absolute values are often not directly comparable even if the technology is the same (30). This could explain the sometimes large variation in threshold estimates observed even when the same technology was used to measure biomarkers. Applying a threshold derived from one cohort to another cohort will flip the +/− labels of participants that are in between the transferred threshold and the cohort’s own threshold. Effectively, this means that with increasing distance between thresholds, more individuals will be assigned to other profiles. Therefore, thresholds could be interchanged with a lower impact on the resulting profiling between ADNI and EPAD, yet, applying PharmaCog’s threshold of tTau on PREVENT-AD would cause considerable disruption of the original PREVENT-AD profiles. While previous studies have found that thresholds were interchangeable across their selected cohort pair (8, 9), our findings raise doubts about the common practice of taking thresholds estimated on a certain dataset to profile participants in a new cohort (31).

### Statistical implications of thresholding method selection

The assumptions made about the data differ vastly across the five evaluated thresholding methods and form their statistical properties. Employing a thresholding method that is more conservative will produce an ATN profiling where fewer individuals will be assigned to profiles with abnormal biomarkers, while less conservative methods will behave oppositely. This links the method selection to the probability of committing type I (i.e., biomarker falsely considered abnormal) and type II (i.e., biomarker falsely considered normal) errors in our resulting profiling and implies that it will inevitably introduce some statistical bias into analyses performed on the achieved ATN profiles. From our experiments, we conclude that such statistical properties are not only of theoretical concern but hugely impact the assignment of participants, as it solely depends on the determined threshold. Therefore, a meaningful comparison of ATN profiles and, consequently, achieved results across studies is likely futile if they relied on different methods.

Furthermore, thresholds themselves are point estimates that are made with uncertainty. We used bootstrapping to assess the uncertainty of our calculated thresholds which revealed relatively stable estimates across most methods and biomarkers. However, in rarer cases, the threshold estimates made from the complete dataset varied substantially from their CIs which raises concerns about the profilings that would result from them. Consequently, careful uncertainty estimation for data-driven thresholds is imperative before employing them in analyses, yet, such uncertainty estimation was seldom done in studies utilizing the ATN framework. Only eleven of the publications we reviewed mentioned some form of uncertainty estimation.

### Results achieved on ATN profiles could generalize beyond the discovery data

A yet underexplored aspect of the ATN framework was the generalizability of results gained on cohort-specific profilings. As thresholds are data-derived, it is possible that not only do their numerical values differ but also the comparability of participants assigned to the same ATN profile is not guaranteed across cohorts.

We observed that participants of all cohorts formed mixed clusters within each respective ATN profile. Only in two profiles, a significant association between cohort membership and cluster assignment was identified, when thresholds were defined using GMM. We believe our results show that ATN-based findings could generalize across different cohorts’ ATN profilings in principle, yet utmost care should be paid here. It remains possible that the generalizability of results could be data modality dependent and might vary for different analysis types such as data-driven prognosis or diagnosis. Conclusively, in an ATN-based setting, it is especially important that results are externally validated in independent data sources while keeping threshold characteristics in mind.

### Limitations

One limitation of our study was that only CSF biomarkers were used for ATN profiling without considering further biomarkers such as PET imaging (1). The reason for this was that they were seldom available in any of the acquired datasets and that we mimicked ATN profiling approaches as they were commonly conducted in previous studies (23, 24, 29). However, previous works have found that fluid and imaging biomarkers are not always concordant (7, 32), thus, it might be that our results are also only CSF biomarker specific. Further, some ATN profiles were subject to low sample sizes, which partially resulted due to the natural progression of AD as well as the already limited number of participants providing CSF samples. Additionally, in a few cohorts, the application of the tertile, ROC, and mean ±2 SD methods was hampered by missing clinical diagnoses. In our clustering analysis assessing participant comparability, we adjusted for systematic differences in MRI variables as, for example, introduced by disparate scanners and protocols across cohort studies. We can not fully rule out that some of these batch effects remained, however, given our observations that cohorts formed mixed clusters, we observed no major influence of cohort membership or batch effects. The set of variables used for the clustering is also limited by the available data and, thus, they do not reflect the full complexity of AD pathology and symptoms. Finally, our previous research has shown that available AD cohorts are hardly representative of the general population as they suffer from inadequate considerations of diversity, equity, and inclusion (33). This might impede the generalizability of our results beyond demographic populations covered in the analyzed datasets, as they predominantly cover participants with a white/caucasian background and recruited their participants exclusively in high-income countries.

We are fully aware that all of the applied thresholding methods have their limitations and make assumptions that are oftentimes not met. We deliberately made the decision to apply them in the way they were commonly used in published ATN-based research to highlight their impact on data analysis. Discussing the statistical intricacies of each approach is beyond the scope of our paper.

## Conclusion

The introduction of the ATN framework constitutes an important step toward a biologically profound definition of AD. However, it is crucial that its intricacies are well understood by researchers in order to generate reliable, robust insights that generalize beyond their discovery data. The results presented in this work highlight that the selection of any specific data-driven thresholding method will inevitably introduce a statistical bias into obtained ATN profiles which will propagate into subsequent analyses.

We want to emphasize that our findings of consistent disease patterns across distinct cohorts’ ATN profiles constitute no ‘carte blanche’ for considering all signals equal across individuals assigned to the same ATN profile. The properties of the data, cohorts’ selection criteria, and employed thresholding method need to be thoroughly investigated whenever data-driven thresholding approaches are used. Establishing harmonized, validated, and potentially experimentally-derived thresholds for each ATN biomarker would circumvent many of the pitfalls in data-driven estimation and substantially improve the generalizability of ATN-based results (28).

### Supplementary Material

Data-driven thresholding statistically biases ATN profiling across cohort datasets

## Data Availability

*Data collection and sharing of ARWIBO was supported by the Italian Ministry of Health, under the following grant agreements:* Ricerca Corrente IRCCS Fatebenefratelli, Linea di Ricerca 2; Progetto Finalizzato Strategico 2000–2001 “Archivio normativo italiano di morfometria cerebrale con risonanza magnetica (età 40+)”; Progetto Finalizzato Strategico 2000–2001 “Decadimento cognitivo lieve non dementigeno: stadio preclinico di malattia di Alzheimer e demenza vascolare. Caratterizzazione clinica, strumentale, genetica e neurobiologica e sviluppo di criteri diagnostici utilizzabili nella realtà nazionale,”; Progetto Finalizzata 2002 “Sviluppo di indicatori di danno cerebrovascolare clinicamente significativo alla risonanza magnetica strutturale”; Progetto Fondazione CARIPLO 2005–2007 “Geni di suscettibilità per gli endofenotipi associati a malattie psichiatriche e dementigene”; “Fitness and Solidarietà”; and anonymous donors. Data used in the preparation of this article were obtained from the Alzheimer’s Disease Repository Without Borders (ARWiBo) (www.arwibo.it). The Principal Investigator of ARWIBO is Giovanni B.Frisoni, MD, University Hospitals and University of Geneva, Geneva, Switzerland, and IRCCS Fatebenefratelli, The National Centre for Alzheimer’s and Mental Diseases, Brescia, Italy. ARWIBO is the result of effort of many researchers of IRCCS Fatebenefratelli: G.Binetti, MD, Neurobiology; L.Bocchio-Chiavetto, PhD, Neuropharmacology; M.Cotelli, PhD, Neuropsychology Unit; C.Minussi, PhD, Neurophysiology; M.Gennarelli, PhD, Genetic Unit; R.Ghidoni, PhD, Proteomics Unit; D.Moretti, MD, and O.Zanetti, MD, Alzheimer’s Unit. EPAD LCS is registered at www.clinicaltrials.gov Identifier: NCT02804789. Data used in preparation of this article were obtained from the EPAD LCS data set V.IMI, doi:10.34688/epadlcs_v.imi_20.10.30. The EPAD LCS was launched in 2015 as a public private partnership, led by Chief Investigator Professor Craig Ritchie MB BS. The primary research goal of the EPAD LCS is to provide a well-phenotyped probability-spectrum population for developing and continuously improving disease models for Alzheimer’s disease in individuals without dementia. This work used data and/or samples from the EPAD project which received support from the EU/EFPIA Innovative Medicines Initiative Joint Undertaking EPAD grant agreement n° 115736 and an Alzheimer’s Association Grant (SG21-818099-EPAD). PharmaCog was funded through the European Community’s ‘Seventh Framework’ Programme (FP7/2007-2013) for an innovative scheme, the Innovative Medicines Initiative (IMI). IMI is a young and unique public-private partnership, founded in 2008 by the pharmaceutical industry (represented by the European Federation of Pharmaceutical Industries and Associations), EFPIA and the European Communities (represented by the European Commission). J-ADNI was supported by the following grants: Translational Research Promotion Project from the New Energy and Industrial Technology Development Organization of Japan; Research on Dementia, Health Labor Sciences Research Grant; Life Science Database Integration Project of Japan Science and Technology Agency; Research Association of Biotechnology (contributed by Astellas Pharma Inc., Bristol-Myers Squibb, Daiichi-Sankyo, Eisai, Eli Lilly and Company, Merck-Banyu, Mitsubishi Tanabe Pharma, Pfizer Inc., Shionogi & Co., Ltd., Sumitomo Dainippon, and Takeda Pharmaceutical Company), Japan, and a grant from an anonymous Foundation. Data collection and sharing for this project was funded by the Alzheimer’s Disease Neuroimaging Initiative (ADNI) (National Institutes of Health Grant U01 AG024904) and DOD ADNI (Department of Defense award number W81XWH-12-2-0012). ADNI is funded by the National Institute on Aging, the National Institute of Biomedical Imaging and Bioengineering, and through generous contributions from the following: AbbVie, Alzheimer’s Association; Alzheimer’s Drug Discovery Foundation; Araclon Biotech; BioClinica, Inc.; Biogen; Bristol-Myers Squibb Company; CereSpir, Inc.; Cogstate; Eisai Inc.; Elan Pharmaceuticals, Inc.; Eli Lilly and Company; EuroImmun; F. Hoffmann-La Roche Ltd and its affiliated company Genentech, Inc.; Fujirebio; GE Healthcare; IXICO Ltd.; Janssen Alzheimer Immunotherapy Research & Development, LLC.; Johnson & Johnson Pharmaceutical Research & Development LLC.; Lumosity; Lundbeck; Merck & Co., Inc.; Meso Scale Diagnostics, LLC.; NeuroRx Research; Neurotrack Technologies; Novartis Pharmaceuticals Corporation; Pfizer Inc.; Piramal Imaging; Servier; Takeda Pharmaceutical Company; and Transition Therapeutics. The Canadian Institutes of Health Research is providing funds to support ADNI clinical sites in Canada. Private sector contributions are facilitated by the Foundation for the National Institutes of Health (www.fnih.org). The grantee organization is the Northern California Institute for Research and Education, and the study is coordinated by the Alzheimer’s Therapeutic Research Institute at the University of Southern California. ADNI data are disseminated by the Laboratory for Neuro Imaging at the University of Southern California. This research was also supported by NIH grants P30 AG010129 and K01 AG030514. The NACC database is funded by NIA/NIH Grant U01 AG016976. NACC data are contributed by the NIA-funded ADCs: P30 AG019610 (PI Eric Reiman, MD), P30 AG013846 (PI Neil Kowall, MD), P30 AG062428-01 (PI James Leverenz, MD) P50 AG008702 (PI Scott Small, MD), P50 AG025688 (PI Allan Levey, MD, PhD), P50 AG047266 (PI Todd Golde, MD, PhD), P30 AG010133 (PI Andrew Saykin, PsyD), P50 AG005146 (PI Marilyn Albert, PhD), P30 AG062421-01 (PI Bradley Hyman, MD, PhD), P30 AG062422-01 (PI Ronald Petersen, MD, PhD), P50 AG005138 (PI Mary Sano, PhD), P30 AG008051 (PI Thomas Wisniewski, MD), P30 AG013854 (PI Robert Vassar, PhD), P30 AG008017 (PI Jeffrey Kaye, MD), P30 AG010161 (PI David Bennett, MD), P50 AG047366 (PI Victor Henderson, MD, MS), P30 AG010129 (PI Charles DeCarli, MD), P50 AG016573 (PI Frank LaFerla, PhD), P30 AG062429-01(PI James Brewer, MD, PhD), P50 AG023501 (PI Bruce Miller, MD), P30 AG035982 (PI Russell Swerdlow, MD), P30 AG028383 (PI Linda Van Eldik, PhD), P30 AG053760 (PI Henry Paulson, MD, PhD), P30 AG010124 (PI John Trojanowski, MD, PhD), P50 AG005133 (PI Oscar Lopez, MD), P50 AG005142 (PI Helena Chui, MD), P30 AG012300 (PI Roger Rosenberg, MD), P30 AG049638 (PI Suzanne Craft, PhD), P50 AG005136 (PI Thomas Grabowski, MD), P30 AG062715-01 (PI Sanjay Asthana, MD, FRCP), P50 AG005681 (PI John Morris, MD), P50 AG047270 (PI Stephen Strittmatter, MD, PhD). *Availability of data and materials:* All datasets used in this work are publicly accessible after successful access application. Links guiding to each individual resource can be found at: https://adata.scai.fraunhofer.de/cohorts.

## Notes

*Ethical standards:* All cohort studies used in this work adhered to the declaration of Helsinki.

## Abbreviations

Aβ1-42: Amyloid-β 1–42
AD: Alzheimer’s disease
ADNI: Alzheimer’s Disease Neuroimaging Initiative
AIBL: Australian Imaging, Biomarker & Lifestyle Flagship Study of Ageing
ARWIBO: Alzheimer’s Disease Repository Without Borders
ATN: Amyloid, Tau, and Neurodegeneration
PET: Positron emission tomography
CDR: Clinical Dementia Rating
CIs: Confidence Intervals
CSF: Cerebrospinal fluid
CU: Cognitively unimpaired
DOD-ADNI: Effects of TBI & PTSD on Alzheimer’s Disease in Vietnam Vets
EDSD: European DTI Study on Dementia
EMIF: European Medical Information Framework
EPAD: European Prevention of Alzheimer’s Dementia
GMMs: Gaussian mixture models
JADNI: Japanese Alzheimer’s Disease Neuroimaging Initiative
MCI: Mild cognitive impairment
MMSE: Mini-Mental State Examination
MRI: Magnetic resonance imaging
NACC: National Alzheimer’s Coordinating Center
NINCDS-ADRDA: National Institute of Neurological and Communicative Diseases and Stroke/Alzheimer’s Disease and Related Disorders Association
PharmaCog: Prediction of Cognitive Properties of New Drug Candidates for Neurodegenerative Diseases in Early Clinical Development
PREVENT-AD: Pre-symptomatic Evaluation of Experimental or Novel Treatments for Alzheimer’s Disease
pTau: Phosphorylated tau
ROC: Receiver operating characteristic
SD: Standard deviation
tTau: Total tau
