# Supplementary material for: Data-Driven Thresholding Statistically Biases ATN Profiling across Cohort Datasets
Source: J Prev Alzheimers Dis. 2023 Sep 6;11(1):185–95. doi: 10.14283/jpad.2023.100 (PMC10995057; doi:10.14283/jpad.2023.100)
Supplement: Supplementary file 1 — Data-driven thresholding statistically biases ATN profiling across cohort datasets [file 42414_2023_100_MOESM1_ESM.docx]

**Supplementary Material: Data-driven thresholding statistically biases ATN profiling across cohort datasets**

Yasamin Salimi^1,2^, Daniel Domingo-Fernández^1^, Martin Hofmann-Apitius^1,2^, and Colin Birkenbihl^1,2^, for the Alzheimer's Disease Neuroimaging Initiative^*^, the Japanese Alzheimer’s Disease Neuroimaging Initiative^†^, the Alzheimer's Disease Repository Without Borders Investigators^‡^, for the European Prevention of Alzheimer’s Disease (EPAD) Consortium^§^

**Author affiliations:**

1. Department of Bioinformatics, Fraunhofer Institute for Algorithms and Scientific Computing (SCAI), Sankt Augustin 53757, Germany
2. Bonn-Aachen International Center for IT, Rheinische Friedrich-Wilhelms-Universität Bonn, Bonn 53115, Germany

Correspondence to: Yasamin Salimi ([yasamin.salimi@scai.fraunhofer.de](mailto:yasamin.salimi@scai.fraunhofer.de)), Colin Birkenbihl ([colin.birkenbihl@scai.fraunhofer.de](mailto:colin.birkenbihl@scai.fraunhofer.de)), Schloß Birlinghoven, Sankt Augustin 53757, Germany

| **Cohort** | **# Patients** | **Age** | **Female %** | **Education** | **APOE4 %** | **CDR** | **MMSE** | **CDRSB** | **Hippocampus** | **tTau** | **pTau** | **Aβ1-42** |
| --- | --- | --- | --- | --- | --- | --- | --- | --- | --- | --- | --- | --- |
| **ADNI [1]** | 1215 | 73.2 (7.3) | 44.7 | 16.0 (2.8) | 46.4 | 0.4 (0.3) | 27.3 (2.6) | 1.6 (1.8) | 6862.9 (1179.1) | 287.0 (132.7) | 27.6 (14.6) | 979.9 (457.1) |
| **EPAD [2]** | 1776 | 65.6 (7.4) | 55.8 | 14.4 (3.7) | 39.5 | 0.1 (0.2) | 28.4 (1.9) | 0.4 (0.7) | 4733.5 (773.0) | 225.8 (99.5) | 19.8 (10.6) | 1216.5 (429.3) |
| **AIBL [3]** | 57 | 73.8 (6.4) | 45.6 | 12.7 (2.8) | 34.5 | 0.3 (0.4) | 26.4 (4.9) | 1.6 (2.7) | 2.8 (0.4) | 438.8 (276.1) | 68.5 (30.5) | 633.0 (241.8) |
| **ARWIBO [4]** | 234 | 70.6 (7.9) | 54.3 | 7.6 (4.0) | 38.5 | 0.7 (0.5) | 23.2 (5.0) | - | 6167.5 (1221.3) | 465.4 (307.3) | 75.1 (50.1) | 506.2 (238.0) |
| **EDSD [5]** | 86 | 70.0 (6.9) | 44.2 | 11.8 (3.0) | 54.7 | - | 26.6 (2.1) | - | 6995.7 (1178.0) | 456.8 (276.8) | 82.0 (40.6) | 665.1 (345.9) |
| **PREVENT-AD [6]** | 133 | 62.8 (5.3) | 67.7 | 15.2 (3.0) | - | - | - | - | - | 284.8 (156.6) | 49.0 (20.3) | 1184.9 (298.3) |
| **PharmaCog [7]** | 145 | 69.2 (7.3) | 57.2 | 10.6 (4.4) | 46.2 | 0.5 (0.0) | 26.6 (1.8) | - | 6729.3 (1420.6) | 475.5 (345.2) | 67.6 (34.7) | 693.0 (292.5) |
| **NACC [8]** | 506 | 67.4 (10.0) | 50.2 | 15.7 (3.1) | 46.6 | 0.4 (0.5) | 26.1 (4.8) | 2.0 (2.6) | 6.4 (0.9) | 235.0 (288.2) | 48.4 (34.9) | 389.9 (254.2) |
| **EMIF [9]** | 1014 | 67.9 (8.6) | 44.8 | 11.2 (4.1) | - | 0.4 (0.3) | 26.1 (3.8) | - | 6956.4 (1212.5) | 377.5 (327.6) | 60.0 (33.6) | 586.4 (282.6) |
| **DOD-ADNI [10]** | 113 | 68.5 (4.2) | 0.9 | 15.2 (2.3) | 25.7 | 0.1 (0.2) | 28.4 (1.5) | 0.4 (0.7) | 7741.6 (970.7) | 219.6 (80.8) | 19.1 (8.3) | 1242.6 (490.9) |
| **JADNI [11]** | 197 | 71.3 (6.8) | 50.8 | 13.4 (2.8) | 47.7 | 0.4 (0.3) | 26.3 (3.0) | 1.8 (1.7) | 6221.8 (1229.4) | 114.1 (61.2) | 56.0 (24.3) | 354.1 (146.5) |

**Table S1:** Summary statistics of participants in each cohort study. Numerical measurements are reported as the mean and standard deviation in parentheses. Categorical variables are presented based on the proportion of participants within a category. Note: APOE4 %, the proportion of participants with at least one APOE e4 status.

| **Cohort** | **# Participants** | **Assay** |
| --- | --- | --- |
| **NACC** | 205 | INNOTEST® kit assay (Innogenetics, Ghent, Belgium), Enzyme-linked immunosorbent assay (ELISA) |
|  | 301 | Multiplex xMAP Luminex platform (LuminexCorp., Austin, TX, USA) with Innogenetics (INNO-BIA AlzBio3, Ghent, Belgium) immunoassay |
| **EMIF** | 811 | INNOTEST® kit assay (Innogenetics, Ghent, Belgium), Enzyme-linked immunosorbent assay (ELISA) |
|  | 203 | Multiplex xMAP Luminex platform (LuminexCorp., Austin, TX, USA) with Innogenetics (INNO-BIA AlzBio3, Ghent, Belgium) immunoassay |

**Table S2:** Number of participants in certain cohorts with CSF measurements using each assay.

| **Cohort** | **Aβ1-42** | **pTau** | **tTau** | **# Patients** |
| --- | --- | --- | --- | --- |
| **ADNI** | <200, >1700 | <8, >120 | <80, >1300 | 204 |
| **ARWIBO** | - | <15.6 | <75, >1200, >1209.5 | 18 |
| **EPAD** | <200, >1700 | <8 | <80 | 531 |

**Table S3:** Technical limits for the CSF assays employed in ADNI, ARWIBO, and EPAD. # Patients, the number of patients with at least one biomarker below or above the technical limit, for which the respective technical limit was taken as a measurement estimate.


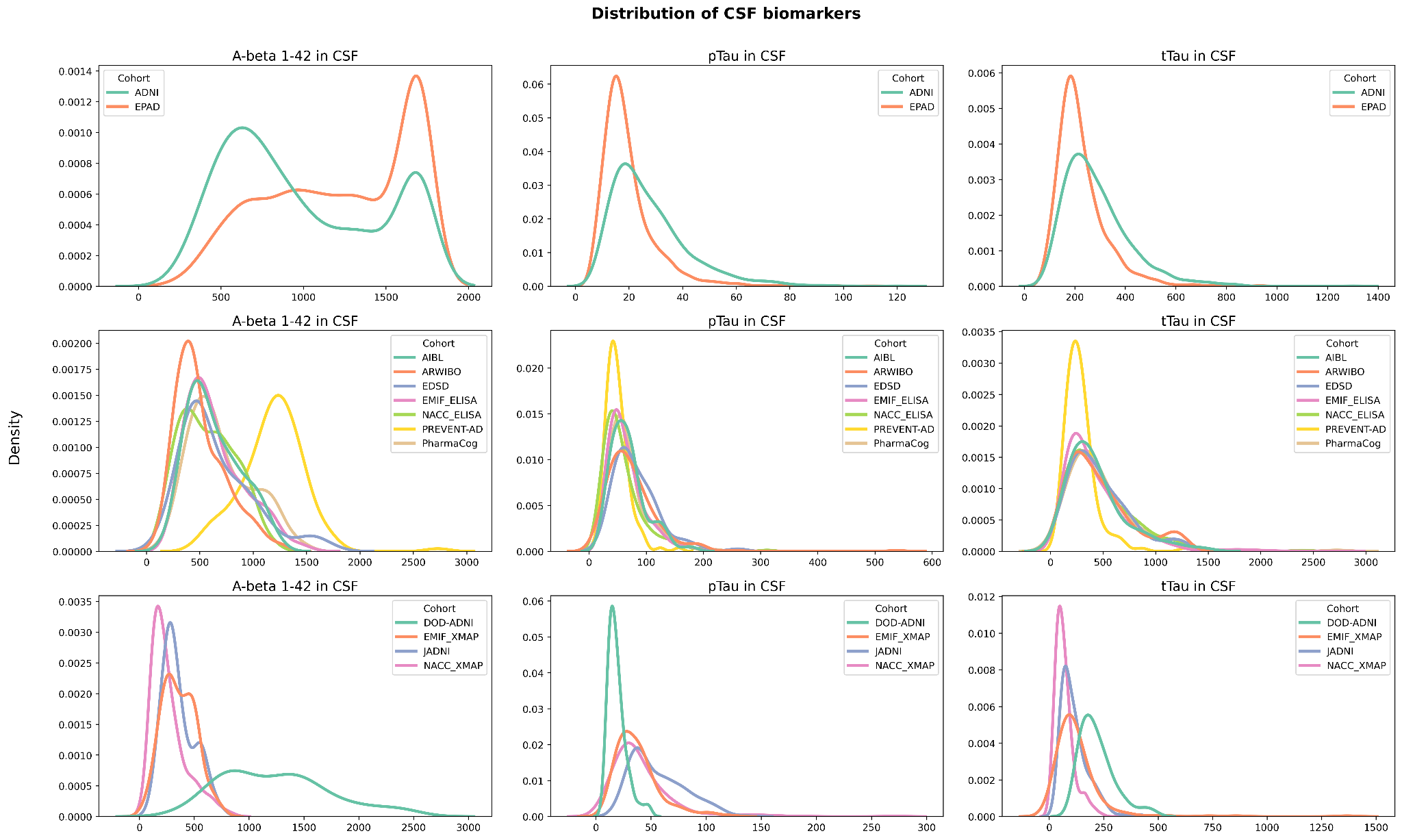


**Figure S1:** The distribution of CSF biomarkers in cohort studies. Note: the cohorts with the same assay method are grouped together in order to compare their distribution.

| **Method** | **Number of Publication** | **Citation** |
| --- | --- | --- |
| **ROC using Youden’s index** | 15 | [12-26] |
| **ROC** | 2 | [27, 28] |
| **ROC using Youden’s index and mean ±2 SD** | 1 | [29] |
| **GMM, Youden’s index and mean ±2 SD** | 1 | [30] |
| **Youden’s index** | 2 | [31-32] |
| **GMM and mean ± SD** | 1 | [33] |
| **GMM and ROC using Youden’s index** | 1 | [34] |
| **GMM and ROC** | 1 | [35] |
| **Sparse K-means** | 1 | [36] |
| **ROC using Youden’s index and regression analysis** | 1 | [37] |
| **Threshold from previous studies, GMM and ROC** | 1 | [38] |
| **Tertile and quartiles** | 1 | [39] |
| **Threshold from previous studies** | 26 | [40-65] |
| **Threshold from previous studies, mean ±2 SD and 90th percentile** | 1 | [66] |
| **Youden’s index and mean ±2 SD** | 1 | [67] |

**Table S4:** Commonly used methodologies extracted from literature for defining thresholds of ATN biomarkers.

| **Common Features** | | | |
| --- | --- | --- | --- |
| Cortical White Matter Volume | Left Pars Triangularis Gray Matter Volume | Right Caudal Anterior Cingulate Mean Cortical Thickness | Right Parsorbitalis Mean Cortical Thickness |
| Left Caudal Anterior Cingulate Mean Cortical Thickness | Left Pars Triangularis Mean Cortical Thickness | Right Cuneus Mean Cortical Thickness | Right Pericalcarine Gray Matter Volume |
| Left Caudal Middle Frontal Mean Cortical Thickness | Left Parsorbitalis Mean Cortical Thickness | Right Entorightinal Mean Cortical Thickness | Right Pericalcarine Mean Cortical Thickness |
| Left Cuneus Mean Cortical Thickness | Left Pericalcarine Gray Matter Volume | Right Fusiform Mean Cortical Thickness | Right Postcentral Grey Matter Volume |
| Left Fusiform Mean Cortical Thickness | Left Pericalcarine Mean Cortical Thickness | Right Hippocampus Volume | Right Postcentral Mean Cortical Thickness |
| Left Hippocampus Volume | Left Postcentral Gray Matter Volume | Right Inferiorparietal Mean Cortical Thickness | Right Posterior Cingulate Gray Matter Volume |
| Left Inferiorparietal Mean Cortical Thickness | Left Postcentral Mean Cortical Thickness | Right Inferiortemporal Mean Cortical Thickness | Right Posterior Cingulate Mean Cortical Thickness |
| Left Inferiortemporal Mean Cortical Thickness | Left Posterior Cingulate Gray Matter Volume | Right Insula Mean Cortical Thickness | Right Precentral Gray Matter Volume |
| Left Insula Mean Cortical Thickness | Left Posterior Cingulate Mean Cortical Thickness | Right Isthmus Cingulate Mean Cortical Thickness | Right Precentral Mean Cortical Thickness |
| Left Isthmus Cingulate Mean Cortical Thickness | Left Precentral Gray Matter Volume | Right Lateral Occipital Gray Matter Volume | Right Precuneus Grey Matter Volume |
| Left Lateral Occipital Gray Matter Volume | Left Precentral Mean Cortical Thickness | Right Lateral Ventricle Volume | Right Precuneus Mean Cortical Thickness |
| Left Lateral Ventricle Volume | Left Precuneus Grey Matter Volume | Right Lateraloccipital Mean Cortical Thickness | Right Rostral Anterior Cingulate Gray Matter Volume |
| Left Lateraloccipital Mean Cortical Thickness | Left Precuneus Mean Cortical Thickness | Right Lateralorbitofrontal Mean Cortical Thickness | Right Rostral Anterior Cingulate Mean Cortical Thickness |
| Left Lateralorbitofrontal Mean Cortical Thickness | Left Rostral Anterior Cingulate Gray Matter Volume | Right Lingual Grey Matter Volume | Right Rostral Middle Frontal Gray Matter Volume |
| Left Lingual Grey Gray Matter Volume | Left Rostral Anterior Cingulate Mean Cortical Thickness | Right Lingual Mean Cortical Thickness | Right Rostral Middle Frontal Mean Cortical Thickness |
| Left Lingual Mean Cortical Thickness | Left Rostral Middle Frontal Gray Matter Volume | Right Medial Orbitofrontal Grey Matter Volume | Right Superior Frontal Gray Matter Volume |
| Left Medial Orbitofrontal Gray Matter Volume | Left Rostral Middle Frontal Mean Cortical Thickness | Right Medial Orbitofrontal Mean Cortical Thickness | Right Superior Parietal Gray Matter Volume |
| Left Medial Orbitofrontal Mean Cortical Thickness | Left Superior Frontal Gray Matter Volume | Right Middle Temporal Gray Matter Volume | Right Superior Temporal Gray Matter Volume |
| Left Middle Temporal Gray Matter Volume | Left Superior Frontal Mean Cortical Thickness | Right Middle Temporal Mean Cortical Thickness | Right Superior Temporal Mean Cortical Thickness |
| Left Middle Temporal Mean Cortical Thickness | Left Superior Parietal Gray Matter Volume | Right Paracentral Mean Cortical Thickness | Right Superiorfrontal Mean Cortical Thickness |
| Left Paracentral Gray Matter Volume | Left Superior Temporal Mean Cortical Thickness | Right Parahippocampal Mean Cortical Thickness | Right Superiorparietal Mean Cortical Thickness |
| Left Paracentral Mean Cortical Thickness | Left Superiorparietal Mean Cortical Thickness | Right Pars Opercularis Gray Matter Volume | Right Supramarginal Gray Matter Volume |
| Left Parahippocampal Mean Cortical Thickness | Left Supramarginal Gray Matter Volume | Right Pars Opercularis Mean Cortical Thickness | Right Supramarginal Mean Cortical Thickness |
| Left Pars Opercularis Gray Matter Volume | Left Supramarginal Mean Cortical Thickness | Right Pars Orbitalis Gray Matter Volume | Right Transverse Temporal Grey Matter Volume |
| Left Pars Opercularis Mean Cortical Thickness | Left Transverse Temporal Grey Matter Volume | Right Pars Triangularis Gray Matter Volume | Right Transverse Temporal Mean Cortical Thickness |
| Left Pars Orbitalis Gray Matter Volume | Left Transverse Temporal Mean Cortical Thickness | Right Pars Triangularis Mean Cortical Thickness | APOE ε4 allele status |
| Third Ventricle Volume | Mini-Mental State Examination (MMSE) |  |  |

**Table S5:** The common attributes among the investigated cohorts that were used for clustering and UMAP analysis. Here, 104 MRI measurements, the APOE ε4 status of participants, and MMSE were utilized.

| **Cohort** | **Method** | | | | | | | | | | | | | | |
| --- | --- | --- | --- | --- | --- | --- | --- | --- | --- | --- | --- | --- | --- | --- | --- |
|  | **GMM** | | | **K-means** | | | **Tertile** | | | **ROC** | | | **Mean ±2 SD** | | |
|  | **Aβ1-42** | **pTau** | **tTau** | **Aβ1-42** | **pTau** | **tTau** | **Aβ1-42** | **pTau** | **tTau** | **Aβ1-42** | **pTau** | **tTau** | **Aβ1-42** | **pTau** | **tTau** |
| **ADNI** | 1185.4 [1179.0, 1191.8] | 35.6 [35.5, 35.7] | 365.8 [364.7, 366.9] | 1094.8 [1094.2, 1095.4] | 27.4 [27.4, 27.5] | 285.4 [285.1, 285.6] | 950.0 [948.2, 951.9] | 23.4 [23.4, 23.5] | 258.8 [258.4, 259.2] | 866.5 [863.8, 869.2] | 24.9 [24.8, 25.1] | 269.0 [268.4, 269.7] | 321.7 [319.4, 324.0] | 40.2 [40.1, 40.3] | 417.8 [417.1, 418.5] |
| **EPAD** | 1382.0 [1381.3, 1382.6] | 29.2 [29.2, 29.3] | 311.6 [311.1, 312.2] | 1176.9 [1176.2, 1177.6] | 19.6 [19.6, 19.7] | 224.7 [224.5, 224.8] | 989.9 [988.9, 991.0] | 20.3 [20.2, 20.3] | 237.6 [237.4, 237.8] | 683.3 [679.1, 687.5] | 24.2 [23.8, 24.6] | 277.8 [272.8, 282.9] | 366.7 [365.6, 367.7] | 40.5 [40.5, 40.6] | 421.5 [420.9, 422.1] |
| **AIBL** | 747.6 [742.0, 753.2] | 87.7 [86.6, 88.7] | 604.1 [596.8, 611.5] | 683.1 [680.3, 685.9] | 76.9 [76.2, 77.6] | 526.2 [519.0, 533.3] | - | - | - | - | - | - | - | - | - |
| **ARWIBO** | 591.7 [588.3, 595.2] | 143.4 [137.1, 149.7] | 587.6 [582.3, 592.9] | 524.1 [521.9, 526.4] | 72.1 [71.8, 72.4] | 572.4 [568.0, 576.9] | - | - | - | - | - | - | - | - | - |
| **EDSD** | 781.9 [775.6, 788.1] | 120.2 [118.7, 121.7] | 592.9 [584.0, 601.8] | 738.4 [732.7, 744.1] | 87.7 [86.9, 88.5] | 549.5 [544.7, 554.2] | - | - | - | - | - | - | - | - | - |
| **PREVENT-AD** | 1202.3 [1172.5, 1232.0] | 70.9 [70.1, 71.8] | 474.6 [464.8, 484.4] | 1144.7 [1142.3, 1147.2] | 50.5 [50.3, 50.7] | 306.8 [304.8, 308.8] | 1097.8 [1095.9, 1099.7] | 53.0 [52.9, 53.1] | 302.8 [301.9, 303.6] | - | - | - | 595.9 [592.0, 599.9] | 89.0 [88.6, 89.4] | 592.2 [588.4, 596.1] |
| **PharmaCog** | 792.4 [790.6, 794.2] | 93.2 [92.9, 93.5] | 778.1 [755.1, 801.1] | 763.2 [760.7, 765.8] | 68.6 [68.0, 69.2] | 465.3 [463.1, 467.5] | - | - | - | - | - | - | - | - | - |
| **NACC_ELISA** | 593.4 [591.4, 595.5] | 81.9 [81.1, 82.7] | 574.0 [559.4, 588.6] | 585.1 [584.0, 586.3] | 61.8 [61.6, 62.1] | 492.3 [490.5, 494.1] | 625.8 [623.9, 627.6] | 48.1 [48.0, 48.2] | 369.6 [367.9, 371.3] | 450.8 [447.5, 454.1] | 61.1 [60.8, 61.5] | 502.8 [498.4, 507.3] | 294.3 [292.4, 296.2] | 87.0 [86.5, 87.5] | 722.5 [719.3, 725.8] |
| **EMIF_ELISA** | 742.2 [741.3, 743.0] | 81.9 [81.8, 82.1] | 525.0 [522.7, 527.3] | 700.6 [698.3, 703.0] | 64.8 [64.7, 64.9] | 432.5 [431.7, 433.3] | 541.4 [540.2, 542.6] | 52.2 [52.1, 52.3] | 268.3 [267.6, 269.1] | 575.7 [571.5, 580.0] | 62.6 [62.1, 63.0] | 336.4 [334.3, 338.6] | 175.2 [173.7, 176.7] | 94.6 [94.0, 95.1] | 649.5 [640.8, 658.1] |
| **NACC_XMAP** | 300.2 [298.5, 302.0] | 60.4 [59.6, 61.1] | 94.3 [93.6, 95.0] | 290.8 [288.5, 293.2] | 40.0 [39.9, 40.1] | 70.1 [69.9, 70.4] | 248.5 [247.7, 249.2] | 36.5 [36.4, 36.5] | 53.7 [53.6, 53.9] | 225.1 [224.5, 225.7] | 40.5 [40.3, 40.7] | 63.0 [62.4, 63.6] | 23.7 [22.7, 24.8] | 68.7 [68.3, 69.1] | 99.1 [98.7, 99.6] |
| **EMIF_XMAP** | 391.5 [389.1, 394.0] | 51.9 [51.5, 52.4] | 229.9 [222.7, 237.0] | 363.1 [362.1, 364.0] | 37.5 [37.4, 37.6] | 129.7 [129.1, 130.2] | 448.7 [447.0, 450.4] | 31.4 [31.1, 31.6] | 80.3 [79.8, 80.9] | 376.9 [374.6, 379.2] | 30.6 [30.4, 30.9] | 98.6 [98.0, 99.1] | 245.6 [242.5, 248.8] | 54.3 [53.9, 54.6] | 122.3 [121.7, 122.8] |
| **DOD-ADNI** | 1412.6 [1395.9, 1429.2] | 26.6 [26.5, 26.7] | 304.2 [302.7, 305.7] | 1280.0 [1273.4, 1286.5] | 22.3 [22.2, 22.4] | 249.9 [248.9, 250.9] | 931.7 [927.8, 935.6] | 19.9 [19.9, 20.0] | 234.2 [233.5, 235.0] | - | - | - | 293.6 [289.7, 297.5] | 34.1 [33.9, 34.2] | 368.2 [367.0, 369.4] |
| **JADNI** | 415.4 [413.4, 417.4] | 61.4 [61.1, 61.7] | 134.1 [132.8, 135.3] | 393.2 [391.3, 395.1] | 59.9 [59.7, 60.1] | 129.8 [128.7, 130.8] | 390.8 [389.0, 392.6] | 38.5 [38.4, 38.6] | 72.2 [71.9, 72.4] | 342.4 [341.0, 343.9] | 46.4 [46.1, 46.6] | 84.9 [84.7, 85.0] | 191.2 [189.4, 193.0] | 65.1 [64.6, 65.6] | 126.3 [125.4, 127.2] |

**Table S6:** Thresholds obtained using each methodology for CSF biomarkers through 1000 bootstraps. The mean thresholds and the confidence intervals, in brackets, are given. The gray cells indicate that the obtained thresholds fell within the confidence intervals.

| **Cohort** | **Method** | | | | | | | | | | | | | | |
| --- | --- | --- | --- | --- | --- | --- | --- | --- | --- | --- | --- | --- | --- | --- | --- |
|  | **GMM** | | | **K-means** | | | **Tertile** | | | **ROC** | | | **Mean ±2 SD** | | |
|  | **Aβ1-42** | **pTau** | **tTau** | **Aβ1-42** | **pTau** | **tTau** | **Aβ1-42** | **pTau** | **tTau** | **Aβ1-42** | **pTau** | **tTau** | **Aβ1-42** | **pTau** | **tTau** |
| **ADNI** | 1.09 % | 0.5 % | 0.59 % | 0.11 % | 0.2 % | 0.18 % | 0.38 % | 0.49 % | 0.31 % | 0.63 % | 0.95 % | 0.5 % | 1.43 % | 0.41 % | 0.34 % |
| **EPAD** | 0.09 % | 0.33 % | 0.34 % | 0.12 % | 0.16 % | 0.14 % | 0.21 % | 0.14 % | 0.16 % | 1.23 % | 3.57 % | 3.67 % | 0.56 % | 0.36 % | 0.27 % |
| **AIBL** | 1.49 % | 2.37 % | 2.44 % | 0.82 % | 1.81 % | 2.72 % | - | - | - | - | - | - | - | - | - |
| **ARWIBO** | 1.17 % | 8.8 % | 1.8 % | 0.86 % | 0.78 % | 1.55 % | - | - | - | - | - | - | - | - | - |
| **EDSD** | 1.6 % | 2.54 % | 3.01 % | 1.54 % | 1.77 % | 1.71 % | - | - | - | - | - | - | - | - | - |
| **PREVENT-AD** | 4.94 % | 2.41 % | 4.13 % | 0.43 % | 0.8 % | 1.3 % | 0.35 % | 0.53 % | 0.54 % | - | - | - | 1.32 % | 0.87 % | 1.29 % |
| **PharmaCog** | 0.45 % | 0.71 % | 5.91 % | 0.67 % | 1.73 % | 0.96 % | - | - | - | - | - | - | - | - | - |
| **NACC_ELISA** | 0.69 % | 1.88 % | 5.08 % | 0.4 % | 0.75 % | 0.74 % | 0.58 % | 0.54 % | 0.92 % | 1.48 % | 1.18 % | 1.78 % | 1.3 % | 1.09 % | 0.9 % |
| **EMIF_ELISA** | 0.24 % | 0.44 % | 0.87 % | 0.68 % | 0.26 % | 0.38 % | 0.46 % | 0.37 % | 0.56 % | 1.47 % | 1.54 % | 1.27 % | 1.75 % | 1.13 % | 2.67 % |
| **NACC_XMAP** | 1.16 % | 2.42 % | 1.49 % | 1.61 % | 0.56 % | 0.65 % | 0.62 % | 0.38 % | 0.59 % | 0.5 % | 1.0 % | 1.79 % | 8.86 % | 1.17 % | 0.98 % |
| **EMIF_XMAP** | 1.25 % | 1.72 % | 6.25 % | 0.53 % | 0.56 % | 0.85 % | 0.76 % | 1.57 % | 1.39 % | 1.23 % | 1.68 % | 1.13 % | 2.56 % | 1.47 % | 0.86 % |
| **DOD-ADNI** | 2.36 % | 0.77 % | 0.97 % | 1.02 % | 0.96 % | 0.79 % | 0.84 % | 0.64 % | 0.64 % | - | - | - | 2.65 % | 0.83 % | 0.66 % |
| **JADNI** | 0.96 % | 0.95 % | 1.89 % | 0.96 % | 0.81 % | 1.64 % | 0.94 % | 0.41 % | 0.71 % | 0.85 % | 1.18 % | 0.43 % | 1.87 % | 1.53 % | 1.47 % |

**Table S7:** The variation among thresholds obtained through bootstrapping for each biomarker using different methodologies.

| **Cohort** | **Biomarkers** | | |
| --- | --- | --- | --- |
|  | **Aβ1-42** | **pTau** | **tTau** |
| **ADNI** | 71.77 | 41.04 | 38.07 |
| **EPAD** | 73.52 | 52.96 | 49.35 |
| **PREVENT-AD** | 48.85 | 43.86 | 49.57 |
| **NACC_ELISA** | 53.66 | 45.45 | 48.33 |
| **EMIF_ELISA** | 76.72 | 45.22 | 59.76 |
| **NACC_xMAP** | 92.7 | 47.62 | 46.04 |
| **EMIF_xMAP** | 46.8 | 48.01 | 59.26 |
| **DOD-ADNI** | 77.87 | 42.27 | 38.09 |
| **JADNI** | 55.12 | 41.24 | 50.48 |
| **Average** | 66.33 | 45.3 | 48.77 |

**Table S8:** The relative change among thresholds achieved through different methods for each biomarker. Percentages are calculated with respect to each cohort’s largest threshold for the respective biomarker. AIBL, ARWIBO, EDSD, and PharmaCog were removed from this assessment as only two out of the five thresholding methods could be performed on them.

| **Groups** | **Biomarkers** | | |
| --- | --- | --- | --- |
|  | **Aβ1-42** | **pTau** | **tTau** |
| **ADNI**  **EPAD** | 11.8 % | 16.55 % | 12.87 % |
| **AIBL**  **ARWIBO**  **EDSD**  **PharmaCog**  **PREVENT-AD**  **NACC_ELISA**  **EMIF_ELISA** | 47.12 % | 21.49 % | 31.96 % |
| **NACC_xMAP**  **EMIF_xMAP**  **DOD-ADNI**  **JADNI** | 71.25 % | 51.23 % | 66.85 % |

**Table S9:** The relative changes of obtained thresholds across the cohort with the same employed assay for each biomarker.

| **Groups** | **Method** | | | | |
| --- | --- | --- | --- | --- | --- |
|  | **GMM** | **K-means** | **Tertile** | **ROC** | **Mean ±2 SD** |
| **ADNI**  **EPAD** | 16.56 % | 19.1 % | 8.69 % | 19.54 % | 4.8 % |
| **AIBL**  **ARWIBO**  **EDSD**  **PharmaCog**  **PREVENT-AD**  **NACC_ELISA**  **EMIF_ELISA** | 40.99 % | 49.12 % | 29.78 % | 15.74 % | 31.99 % |
| **NACC_xMAP**  **EMIF_xMAP**  **DOD-ADNI**  **JADNI** | 67.55 % | 71.26 % | 66.4 % | 38.39 % | 71.95 % |

**Table S10:** The relative changes of obtained thresholds across the cohort with the same employed assay within each method.

| **Cohort** | **ATN Biomarker Profile** | | | | | | | |
| --- | --- | --- | --- | --- | --- | --- | --- | --- |
|  | **A-T-N-** | **A-T+N+** | **A-T-N+** | **A-T+N-** | **A+T+N-** | **A+T-N-** | **A+T-N+** | **A+T+N+** |
| **ADNI** | 386 | 21 | 15 | 0 | 32 | 528 | 11 | 222 |
| **EPAD** | 671 | 54 | 26 | 1 | 12 | 834 | 10 | 168 |
| **AIBL** | 17 | 0 | 2 | 0 | 0 | 28 | 1 | 9 |
| **ARWIBO** | 59 | 3 | 9 | 1 | 4 | 93 | 45 | 20 |
| **EDSD** | 20 | 1 | 5 | 1 | 2 | 35 | 10 | 11 |
| **PREVENT-AD** | 89 | 9 | 0 | 3 | 0 | 26 | 1 | 5 |
| **PharmaCog** | 46 | 1 | 2 | 0 | 2 | 69 | 3 | 22 |
| **NACC** | 168 | 6 | 9 | 3 | 12 | 193 | 51 | 64 |
| **EMIF** | 293 | 16 | 10 | 8 | 40 | 422 | 62 | 163 |
| **DOD-ADNI** | 46 | 8 | 0 | 1 | 0 | 50 | 0 | 8 |
| **JADNI** | 55 | 0 | 1 | 0 | 20 | 65 | 5 | 51 |

**Table S11:** The number of categorized participants in each ATN profile using thresholds that were obtained by GMM methodology.

| **Cohort** | **ATN Biomarker Profile** | | | | | | | |
| --- | --- | --- | --- | --- | --- | --- | --- | --- |
|  | **A-T-N-** | **A-T+N+** | **A-T-N+** | **A-T+N-** | **A+T+N-** | **A+T-N-** | **A+T-N+** | **A+T+N+** |
| **ADNI** | 337 | 76 | 27 | 1 | 27 | 347 | 12 | 388 |
| **EPAD** | 608 | 301 | 55 | 6 | 24 | 460 | 7 | 315 |
| **AIBL** | 17 | 1 | 1 | 3 | 3 | 24 | 0 | 8 |
| **ARWIBO** | 61 | 10 | 3 | 14 | 27 | 65 | 5 | 49 |
| **EDSD** | 19 | 3 | 1 | 2 | 11 | 29 | 2 | 18 |
| **PREVENT-AD** | 39 | 30 | 3 | 6 | 2 | 39 | 1 | 13 |
| **PharmaCog** | 40 | 5 | 4 | 0 | 9 | 41 | 3 | 43 |
| **NACC** | 144 | 23 | 13 | 24 | 28 | 123 | 47 | 104 |
| **EMIF** | 265 | 42 | 16 | 35 | 64 | 283 | 37 | 272 |
| **DOD-ADNI** | 36 | 18 | 4 | 0 | 1 | 42 | 3 | 9 |
| **JADNI** | 56 | 0 | 1 | 0 | 29 | 67 | 2 | 42 |

**Table S12:** The number of categorized participants in each ATN profile using thresholds that were obtained by the K-means methodology.

| **Cohort** | **ATN Biomarker Profile** | | | | | | | |
| --- | --- | --- | --- | --- | --- | --- | --- | --- |
|  | **A-T-N-** | **A-T+N+** | **A-T-N+** | **A-T+N-** | **A+T+N-** | **A+T-N-** | **A+T-N+** | **A+T+N+** |
| **ADNI** | 341 | 159 | 19 | 8 | 37 | 226 | 2 | 423 |
| **EPAD** | 813 | 309 | 41 | 14 | 34 | 320 | 5 | 240 |
| **PREVENT-AD** | 52 | 29 | 3 | 5 | 1 | 31 | 2 | 10 |
| **NACC** | 109 | 56 | 19 | 25 | 10 | 88 | 53 | 146 |
| **EMIF** | 207 | 220 | 63 | 29 | 13 | 118 | 46 | 318 |
| **DOD-ADNI** | 46 | 27 | 1 | 1 | 3 | 24 | 2 | 9 |
| **JADNI** | 32 | 19 | 9 | 2 | 11 | 14 | 8 | 102 |

**Table S13:** The number of categorized participants in each ATN profile using thresholds that were obtained by tertile methodology.

| **Cohort** | **ATN Biomarker Profile** | | | | | | | |
| --- | --- | --- | --- | --- | --- | --- | --- | --- |
|  | **A-T-N-** | **A-T+N+** | **A-T-N+** | **A-T+N-** | **A+T+N-** | **A+T-N-** | **A+T-N+** | **A+T+N+** |
| **ADNI** | 379 | 170 | 17 | 9 | 37 | 217 | 2 | 384 |
| **EPAD** | 848 | 502 | 95 | 4 | 13 | 147 | 3 | 164 |
| **NACC** | 182 | 40 | 32 | 11 | 7 | 65 | 67 | 102 |
| **EMIF** | 294 | 148 | 39 | 53 | 22 | 148 | 27 | 283 |
| **JADNI** | 61 | 19 | 9 | 0 | 10 | 16 | 5 | 77 |

**Table S14:** The number of categorized participants in each ATN profile using thresholds that were obtained by ROC (Youdnen’s index) methodology.

| **Cohort** | **ATN Biomarker Profile** | | | | | | | |
| --- | --- | --- | --- | --- | --- | --- | --- | --- |
|  | **A-T-N-** | **A-T+N+** | **A-T-N+** | **A-T+N-** | **A+T+N-** | **A+T-N-** | **A+T-N+** | **A+T+N+** |
| **ADNI** | 989 | 154 | 12 | 33 | 1 | 23 | 0 | 3 |
| **EPAD** | 1663 | 66 | 15 | 11 | 0 | 21 | 0 | 0 |
| **PREVENT-AD** | 123 | 5 | 1 | 1 | 0 | 3 | 0 | 0 |
| **NACC** | 385 | 35 | 43 | 13 | 2 | 17 | 2 | 9 |
| **EMIF** | 742 | 117 | 70 | 33 | 1 | 31 | 15 | 5 |
| **DOD-ADNI** | 106 | 5 | 1 | 1 | 0 | 0 | 0 | 0 |
| **JADNI** | 119 | 48 | 8 | 4 | 3 | 10 | 1 | 4 |

**Table S15:** The number of categorized participants in each ATN profile using thresholds that were obtained by mean ±2 SD methodology.

| **Cohorts** | **GMM** | | | | | | | |
| --- | --- | --- | --- | --- | --- | --- | --- | --- |
|  | **A-T-N-** | **A-T+N+** | **A-T-N+** | **A+T+N-** | **A+T-N-** | **A+T-N+** | **A+T+N+** | **Total** |
| **ADNI** | 263 | 16 | 7 | 17 | 307 | 7 | 119 | 736 |
| **EDSD** | 12 | 0 | 4 | 1 | 26 | 9 | 10 | 62 |
| **ARWIBO** | 24 | 2 | 5 | 2 | 29 | 16 | 11 | 89 |
| **NACC** | 75 | 2 | 7 | 1 | 62 | 27 | 17 | 191 |
| **JADNI** | 52 | 0 | 1 | 17 | 63 | 5 | 49 | 187 |
| **DOD-ADNI** | 28 | 4 | 0 | 0 | 34 | 0 | 4 | 70 |
| **PharmaCog** | 46 | 1 | 2 | 2 | 67 | 3 | 22 | 143 |
| **Total** | 500 | 25 | 26 | 40 | 588 | 67 | 232 | 1478 |

**Table S16:** The number of participants with available MRI measurements, MMSE, and APOE ε4 status included in the ATN-based clustering analysis in each cohort using GMM thresholds.

| **Cohorts** | **K-means** | | | | | | | |
| --- | --- | --- | --- | --- | --- | --- | --- | --- |
|  | **A-T-N-** | **A-T+N+** | **A-T-N+** | **A+T+N-** | **A+T-N-** | **A+T-N+** | **A+T+N+** | **Total** |
| **ADNI** | 231 | 49 | 18 | 15 | 209 | 5 | 208 | 735 |
| **EDSD** | 12 | 2 | 0 | 8 | 21 | 2 | 16 | 61 |
| **ARWIBO** | 21 | 6 | 2 | 13 | 20 | 2 | 19 | 83 |
| **NACC** | 70 | 9 | 2 | 3 | 48 | 21 | 34 | 187 |
| **JADNI** | 53 | 0 | 1 | 26 | 65 | 2 | 40 | 187 |
| **DOD-ADNI** | 23 | 7 | 3 | 1 | 31 | 0 | 5 | 70 |
| **PharmaCog** | 40 | 5 | 4 | 9 | 40 | 3 | 42 | 143 |
| **Total** | 450 | 78 | 30 | 75 | 434 | 35 | 364 | 1466 |

**Table S17:** The number of participants with available MRI measurements, MMSE, and APOE ε4 status included in the ATN-based clustering analysis in each cohort using K-means thresholds.


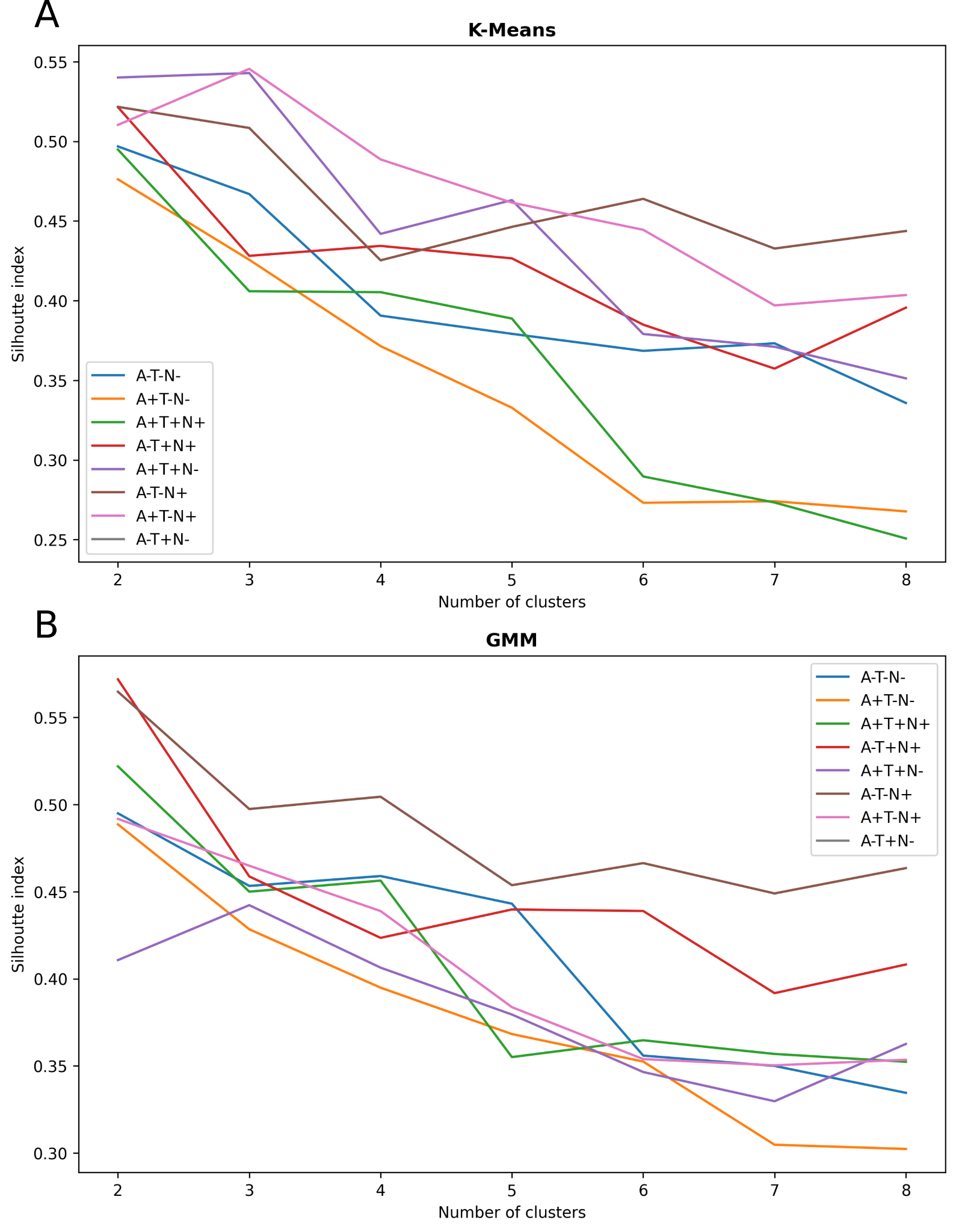


**Figure S2:** Silhouette index for different numbers of clusters within each ATN profile using GMM and K-means thresholds to determine the optimal number of clusters for each profile. The silhouette index measures the distance of data points to their own cluster and contrasts it against the distance to other clusters. The greater the index, the more clearly clusters are separated from each other, with each data point being closer to its assigned cluster than to members of other clusters. A) Using K-means thresholds for the categorization of participants. B) Using GMM thresholds for the categorization of participants.

| **Method** | | **Biomarker Profile** | | | | | | |
| --- | --- | --- | --- | --- | --- | --- | --- | --- |
|  |  | **A-T-N-** | **A-T+N+** | **A-T-N+** | **A+T+N-** | **A+T-N-** | **A+T-N+** | **A+T+N+** |
| **K-means** | **Cramer’s V** | 0.08 | 0.15 | 0.24 | 0.15 | 0.1 | 0 | 0.13 |
|  | ***p*-value** | 0.64 | 0.92 | 0.09 | 0.42 | 0.11 | 0.87 | 0.16 |
|  | **Total Participants** | 450 | 78 | 30 | 75 | 434 | 35 | 364 |
| **GMM** | **Cramer’s V** | 0.1 | 0.09 | 0.14 | 0.36 | 0.09 | 0.19 | 0.16 |
|  | ***p*-value** | 0.26 | 0.94 | 0.16 | 0.01 | 0.06 | 0.1 | 0.02 |
|  | **Total Participants** | 500 | 25 | 26 | 40 | 588 | 67 | 232 |

**Table S18:** Cramer’s V (a measure of association between cohort membership and cluster assignment with 0 meaning no association and 1 perfect association) and the p-value for each clustering of participants in each ATN profile using certain data-driven thresholds. These results are from the clustering experiment in which the number of clusters was set to be equal to the number of cohorts.

##

##

## **References**

1. Mueller SG, Weiner MW, Thal LJ, Petersen RC, Jack CR, Jagust W, et al. Ways toward an early diagnosis in Alzheimer’s disease: the Alzheimer’s Disease Neuroimaging Initiative (ADNI). Alzheimers Dement. 2005;1(1):55-66.
2. Solomon A, Kivipelto M, Molinuevo JL, Tom B, Ritchie CW; EPAD Consortium. European Prevention of Alzheimer's Dementia Longitudinal Cohort Study (EPAD LCS): study protocol. BMJ Open. 2019;8(12):e021017.
3. Ellis KA, Bush AI, Darby D, De Fazio D, Foster J, Hudson P, et al. The Australian Imaging, Biomarkers and Lifestyle (AIBL) study of aging: methodology and baseline characteristics of 1112 individuals recruited for a longitudinal study of Alzheimer's disease. Int Psychogeriatr. 2009;21(4):672-687.
4. Frisoni GB, Prestia A, Zanetti O, Galluzzi S, Romano M, Cotelli M, et al. Markers of Alzheimer's disease in a population attending a memory clinic. Alzheimers Dement. 2009;5(4):307-317.
5. Brueggen K, Grothe MJ, Dyrba M, Fellgiebel A, Fischer F, Filippi M, et al.The European DTI Study on Dementia - A multicenter DTI and MRI study on Alzheimer's disease and Mild Cognitive Impairment. Neuroimage. 2017;144(Pt B):305-308.
6. Breitner JCS, Poirier J, Etienne PE, Leoutsakos JM. Rationale and Structure for a New Center for Studies on Prevention of Alzheimer's Disease (StoP-AD). J Prev Alzheimers Dis. 2016;3(4):236-242.
7. Galluzzi S, Marizzoni M, Babiloni C, Albani D, Antelmi L, Bagnoli C, et al. Clinical and biomarker profiling of prodromal Alzheimer's disease in workpackage 5 of the Innovative Medicines Initiative PharmaCog project: a 'European ADNI study'. J Intern Med. 2016;279(6):576-591.
8. Besser L, Kukull W, Knopman DS, Chui H, Galasko D, Weintraub S, et al. Version 3 of the National Alzheimer’s Coordinating Center’s Uniform Data Set. Alzheimer Dis Assoc Disord. 2018;32(4):351-358.
9. Bos I, Vos S, Vandenberghe R, Scheltens P, Engelborghs S, Frisoni G, et al. The EMIF-AD Multimodal Biomarker Discovery study: design, methods and cohort characteristics. Alzheimers Res Ther. 2018;10(1):64.
10. Weiner MW, Veitch DP, Hayes J, Neylan T, Grafman J, Aisen PS, et al. Effects of traumatic brain injury and posttraumatic stress disorder on Alzheimer's disease in veterans, using the Alzheimer's Disease Neuroimaging Initiative. Alzheimers Dement. 2014;10(3 Suppl):S226-S235.
11. Iwatsubo T. Japanese Alzheimer's Disease Neuroimaging Initiative: present status and future. Alzheimers Dement. 2010;6(3):297-299.
12. Shiino, A., Shirakashi, Y., Ishida, M., Tanigaki, K., & Japanese Alzheimer's Disease Neuroimaging Initiative (2021). Machine learning of brain structural biomarkers for Alzheimer's disease (AD) diagnosis, prediction of disease progression, and amyloid beta deposition in the Japanese population. Alzheimer's & dementia (Amsterdam, Netherlands), 13(1), e12246. <https://doi.org/10.1002/dad2.12246>
13. Song, R., Wu, X., Liu, H., Guo, D., Tang, L., Zhang, W., Feng, J., & Li, C. (2022). Prediction of Cognitive Progression in Individuals with Mild Cognitive Impairment Using Radiomics as an Improvement of the ATN System: A Five-Year Follow-Up Study. Korean journal of radiology, 23(1), 89–100. <https://doi.org/10.3348/kjr.2021.0323>
14. Lee, J., Jang, H., Kang, S. H., Kim, J., Kim, J. S., Kim, J. P., Kim, H. J., Seo, S. W., & Na, D. L. (2020). Cerebrospinal Fluid Biomarkers for the Diagnosis and Classification of Alzheimer's Disease Spectrum. Journal of Korean medical science, 35(44), e361. <https://doi.org/10.3346/jkms.2020.35.e361>
15. Schindler, S. E., Gray, J. D., Gordon, B. A., Xiong, C., Batrla-Utermann, R., Quan, M., Wahl, S., Benzinger, T., Holtzman, D. M., Morris, J. C., & Fagan, A. M. (2018). Cerebrospinal fluid biomarkers measured by Elecsys assays compared to amyloid imaging. Alzheimer's & dementia : the journal of the Alzheimer's Association, 14(11), 1460–1469. <https://doi.org/10.1016/j.jalz.2018.01.013>
16. Provost, K., Iaccarino, L., Soleimani-Meigooni, D. N., Baker, S., Edwards, L., Eichenlaub, U., Hansson, O., Jagust, W., Janabi, M., La Joie, R., Lesman-Segev, O., Mellinger, T. J., Miller, B. L., Ossenkoppele, R., Pham, J., Smith, R., Sonni, I., Strom, A., Mattsson-Carlgren, N., Rabinovici, G. D., … Alzheimer’s Disease Neuroimaging Initiative (ADNI) (2021). Comparing ATN-T designation by tau PET visual reads, tau PET quantification, and CSF PTau181 across three cohorts. European journal of nuclear medicine and molecular imaging, 48(7), 2259–2271. <https://doi.org/10.1007/s00259-020-05152-8>
17. Delmotte, K., Schaeverbeke, J., Poesen, K., & Vandenberghe, R. (2021). Prognostic value of amyloid/tau/neurodegeneration (ATN) classification based on diagnostic cerebrospinal fluid samples for Alzheimer's disease. Alzheimer's research & therapy, 13(1), 84. <https://doi.org/10.1186/s13195-021-00817-4>
18. Cousins, K., Phillips, J. S., Irwin, D. J., Lee, E. B., Wolk, D. A., Shaw, L. M., Zetterberg, H., Blennow, K., Burke, S. E., Kinney, N. G., Gibbons, G. S., McMillan, C. T., Trojanowski, J. Q., & Grossman, M. (2021). ATN incorporating cerebrospinal fluid neurofilament light chain detects frontotemporal lobar degeneration. Alzheimer's & dementia : the journal of the Alzheimer's Association, 17(5), 822–830. <https://doi.org/10.1002/alz.12233>
19. Blazhenets, G., Frings, L., Ma, Y., Sörensen, A., Eidelberg, D., Wiltfang, J., Meyer, P. T., & Alzheimer's Disease Neuroimaging Initiative (2021). Validation of the Alzheimer Disease Dementia Conversion-Related Pattern as an ATN Biomarker of Neurodegeneration. Neurology, 96(9), e1358–e1368. <https://doi.org/10.1212/WNL.0000000000011521>
20. Guo, Y., Li, H. Q., Tan, L., Chen, S. D., Yang, Y. X., Ma, Y. H., Zuo, C. T., Dong, Q., Tan, L., Yu, J. T., & Alzheimer's Disease Neuroimaging Initiative (2020). Discordant Alzheimer's neurodegenerative biomarkers and their clinical outcomes. Annals of clinical and translational neurology, 7(10), 1996–2009. <https://doi.org/10.1002/acn3.51196>
21. Torso, M., Bozzali, M., Zamboni, G., Jenkinson, M., Chance, S. A., & Alzheimers Disease Neuroimage Initiative (2021). Detection of Alzheimer's Disease using cortical diffusion tensor imaging. Human brain mapping, 42(4), 967–977. <https://doi.org/10.1002/hbm.25271>
22. Aksnes, M., Tiiman, A., Edwin, T. H., Terenius, L., Bogdanović, N., Vukojević, V., & Knapskog, A. B. (2021). Comparison of Cerebrospinal Fluid Amyloidogenic Nanoplaques With Core Biomarkers of Alzheimer's Disease. Frontiers in aging neuroscience, 12, 608628. <https://doi.org/10.3389/fnagi.2020.608628>
23. Illán-Gala, I., Pegueroles, J., Montal, V., Vilaplana, E., Carmona-Iragui, M., Alcolea, D., Dickerson, B. C., Sánchez-Valle, R., de Leon, M. J., Blesa, R., Lleó, A., & Fortea, J. (2018). Challenges associated with biomarker-based classification systems for Alzheimer's disease. Alzheimer's & dementia (Amsterdam, Netherlands), 10, 346–357. <https://doi.org/10.1016/j.dadm.2018.03.004>
24. Hansen, E. O., Dias, N. S., Burgos, I., Costa, M. V., Carvalho, A. T., Teixeira, A. L., Barbosa, I. G., Santos, L., Rosa, D., Ribeiro, A., Viana, B. M., & Bicalho, M. (2021). Millipore xMap® Luminex (HATMAG-68K): An Accurate and Cost-Effective Method for Evaluating Alzheimer's Biomarkers in Cerebrospinal Fluid. Frontiers in psychiatry, 12, 716686. <https://doi.org/10.3389/fpsyt.2021.716686>
25. Shen, X. N., Li, J. Q., Wang, H. F., Li, H. Q., Huang, Y. Y., Yang, Y. X., Tan, L., Dong, Q., Yu, J. T., & Alzheimer's Disease Neuroimaging Initiative (2020). Plasma amyloid, tau, and neurodegeneration biomarker profiles predict Alzheimer's disease pathology and clinical progression in older adults without dementia. Alzheimer's & dementia (Amsterdam, Netherlands), 12(1), e12104. <https://doi.org/10.1002/dad2.12104>
26. Hwang, J., Jeong, J. H., Yoon, S. J., Park, K. W., Kim, E. J., Yoon, B., Jang, J. W., Kim, H. J., Hong, J. Y., Lee, J. M., Park, H., Kang, J. H., Choi, Y. H., Park, G., Hong, J., Byun, M. S., Yi, D., Kim, Y. K., Lee, D. Y., & Choi, S. H. (2019). Clinical and Biomarker Characteristics According to Clinical Spectrum of Alzheimer's Disease (AD) in the Validation Cohort of Korean Brain Aging Study for the Early Diagnosis and Prediction of AD. Journal of clinical medicine, 8(3), 341. <https://doi.org/10.3390/jcm8030341>
27. Shaw, L. M., Vanderstichele, H., Knapik-Czajka, M., Clark, C. M., Aisen, P. S., Petersen, R. C., Blennow, K., Soares, H., Simon, A., Lewczuk, P., Dean, R., Siemers, E., Potter, W., Lee, V. M., Trojanowski, J. Q., & Alzheimer's Disease Neuroimaging Initiative (2009). Cerebrospinal fluid biomarker signature in Alzheimer's disease neuroimaging initiative subjects. Annals of neurology, 65(4), 403–413. <https://doi.org/10.1002/ana.21610>
28. Smirnov, D. S., Ashton, N. J., Blennow, K., Zetterberg, H., Simrén, J., Lantero-Rodriguez, J., Karikari, T. K., Hiniker, A., Rissman, R. A., Salmon, D. P., & Galasko, D. (2022). Plasma biomarkers for Alzheimer's Disease in relation to neuropathology and cognitive change. Acta neuropathologica, 143(4), 487–503. <https://doi.org/10.1007/s00401-022-02408-5>
29. Ossenkoppele, R., Rabinovici, G. D., Smith, R., Cho, H., Schöll, M., Strandberg, O., Palmqvist, S., Mattsson, N., Janelidze, S., Santillo, A., Ohlsson, T., Jögi, J., Tsai, R., La Joie, R., Kramer, J., Boxer, A. L., Gorno-Tempini, M. L., Miller, B. L., Choi, J. Y., Ryu, Y. H., … Hansson, O. (2018). Discriminative Accuracy of [18F]flortaucipir Positron Emission Tomography for Alzheimer Disease vs Other Neurodegenerative Disorders. JAMA, 320(11), 1151–1162. <https://doi.org/10.1001/jama.2018.12917>
30. Mattsson-Carlgren, N., Leuzy, A., Janelidze, S., Palmqvist, S., Stomrud, E., Strandberg, O., Smith, R., & Hansson, O. (2020). The implications of different approaches to define AT(N) in Alzheimer disease. Neurology, 94(21), e2233–e2244. <https://doi.org/10.1212/WNL.0000000000009485>
31. Cullen, N. C., Leuzy, A., Janelidze, S., Palmqvist, S., Svenningsson, A. L., Stomrud, E., Dage, J. L., Mattsson-Carlgren, N., & Hansson, O. (2021). Plasma biomarkers of Alzheimer's disease improve prediction of cognitive decline in cognitively unimpaired elderly populations. Nature communications, 12(1), 3555. <https://doi.org/10.1038/s41467-021-23746-0>
32. Seo, E. H., Lim, H. J., Yoon, H. J., Choi, K. Y., Lee, J. J., Park, J. Y., Choi, S. H., Kim, H., Kim, B. C., & Lee, K. H. (2021). Visuospatial memory impairment as a potential neurocognitive marker to predict tau pathology in Alzheimer's continuum. Alzheimer's research & therapy, 13(1), 167. <https://doi.org/10.1186/s13195-021-00909-1>
33. Calvin, C. M., de Boer, C., Raymont, V., Gallacher, J., Koychev, I., & European Prevention of Alzheimer’s Dementia (EPAD) Consortium (2020). Prediction of Alzheimer's disease biomarker status defined by the 'ATN framework' among cognitively healthy individuals: results from the EPAD longitudinal cohort study. Alzheimer's research & therapy, 12(1), 143. <https://doi.org/10.1186/s13195-020-00711-5>
34. Altomare, D., de Wilde, A., Ossenkoppele, R., Pelkmans, W., Bouwman, F., Groot, C., van Maurik, I., Zwan, M., Yaqub, M., Barkhof, F., van Berckel, B. N., Teunissen, C. E., Frisoni, G. B., Scheltens, P., & van der Flier, W. M. (2019). Applying the ATN scheme in a memory clinic population: The ABIDE project. Neurology, 93(17), e1635–e1646. <https://doi.org/10.1212/WNL.0000000000008361>
35. Hansson, O., Seibyl, J., Stomrud, E., Zetterberg, H., Trojanowski, J. Q., Bittner, T., Lifke, V., Corradini, V., Eichenlaub, U., Batrla, R., Buck, K., Zink, K., Rabe, C., Blennow, K., Shaw, L. M., Swedish BioFINDER study group, & Alzheimer's Disease Neuroimaging Initiative (2018). CSF biomarkers of Alzheimer's disease concord with amyloid-β PET and predict clinical progression: A study of fully automated immunoassays in BioFINDER and ADNI cohorts. Alzheimer's & dementia : the journal of the Alzheimer's Association, 14(11), 1470–1481. <https://doi.org/10.1016/j.jalz.2018.01.010>
36. Cohen, A. D., Mowrey, W., Weissfeld, L. A., Aizenstein, H. J., McDade, E., Mountz, J. M., Nebes, R. D., Saxton, J. A., Snitz, B., Dekosky, S., Williamson, J., Lopez, O. L., Price, J. C., Mathis, C. A., & Klunk, W. E. (2013). Classification of amyloid-positivity in controls: comparison of visual read and quantitative approaches. NeuroImage, 71, 207–215. <https://doi.org/10.1016/j.neuroimage.2013.01.015>
37. Bellomo, G., Cataldi, S., Paciotti, S., Paolini Paoletti, F., Chiasserini, D., & Parnetti, L. (2020). Measurement of CSF core Alzheimer disease biomarkers for routine clinical diagnosis: do fresh vs frozen samples differ?. Alzheimer's research & therapy, 12(1), 121. <https://doi.org/10.1186/s13195-020-00689-0>
38. Ingala, S., De Boer, C., Masselink, L. A., Vergari, I., Lorenzini, L., Blennow, K., Chételat, G., Di Perri, C., Ewers, M., van der Flier, W. M., Fox, N. C., Gispert, J. D., Haller, S., Molinuevo, J. L., Muniz-Terrera, G., Mutsaerts, H. J., Ritchie, C. W., Ritchie, K., Schmidt, M., Schwarz, A. J., … EPAD consortium (2021). Application of the ATN classification scheme in a population without dementia: Findings from the EPAD cohort. Alzheimer's & dementia : the journal of the Alzheimer's Association, 17(7), 1189–1204. <https://doi.org/10.1002/alz.12292>
39. Soldan, A., Pettigrew, C., Fagan, A. M., Schindler, S. E., Moghekar, A., Fowler, C., Li, Q. X., Collins, S. J., Carlsson, C., Asthana, S., Masters, C. L., Johnson, S., Morris, J. C., Albert, M., & Gross, A. L. (2019). ATN profiles among cognitively normal individuals and longitudinal cognitive outcomes. Neurology, 92(14), e1567–e1579. <https://doi.org/10.1212/WNL.0000000000007248>
40. Tan, M. S., Ji, X., Li, J. Q., Xu, W., Wang, H. F., Tan, C. C., Dong, Q., Zuo, C. T., Tan, L., Suckling, J., Yu, J. T., & Alzheimer’s Disease Neuroimaging Initiative (2020). Longitudinal trajectories of Alzheimer's ATN biomarkers in elderly persons without dementia. Alzheimer's research & therapy, 12(1), 55. <https://doi.org/10.1186/s13195-020-00621-6>
41. Cousins, K., Irwin, D. J., Wolk, D. A., Lee, E. B., Shaw, L., Trojanowski, J. Q., Da Re, F., Gibbons, G. S., Grossman, M., & Phillips, J. S. (2020). ATN status in amnestic and non-amnestic Alzheimer's disease and frontotemporal lobar degeneration. Brain : a journal of neurology, 143(7), 2295–2311. <https://doi.org/10.1093/brain/awaa165>
42. Ezzati, A., Abdulkadir, A., Jack, C. R., Jr, Thompson, P. M., Harvey, D. J., Truelove-Hill, M., Sreepada, L. P., Davatzikos, C., Alzheimer's Disease Neuroimaging Initiative, & Lipton, R. B. (2021). Predictive value of ATN biomarker profiles in estimating disease progression in Alzheimer's disease dementia. Alzheimer's & dementia : the journal of the Alzheimer's Association, 17(11), 1855–1867. <https://doi.org/10.1002/alz.12491>
43. Nordengen, K., Kirsebom, B. E., Henjum, K., Selnes, P., Gísladóttir, B., Wettergreen, M., Torsetnes, S. B., Grøntvedt, G. R., Waterloo, K. K., Aarsland, D., Nilsson, L., & Fladby, T. (2019). Glial activation and inflammation along the Alzheimer's disease continuum. Journal of neuroinflammation, 16(1), 46. <https://doi.org/10.1186/s12974-019-1399-2>
44. Cedres, N., Ekman, U., Poulakis, K., Shams, S., Cavallin, L., Muehlboeck, S., Granberg, T., Wahlund, L. O., Ferreira, D., Westman, E., & Alzheimer’s Disease Neuroimaging Initiative (2020). Brain Atrophy Subtypes and the ATN Classification Scheme in Alzheimer's Disease. Neuro-degenerative diseases, 20(4), 153–164. <https://doi.org/10.1159/000515322>
45. Karikari, T. K., Emeršič, A., Vrillon, A., Lantero-Rodriguez, J., Ashton, N. J., Kramberger, M. G., Dumurgier, J., Hourregue, C., Čučnik, S., Brinkmalm, G., Rot, U., Zetterberg, H., Paquet, C., & Blennow, K. (2021). Head-to-head comparison of clinical performance of CSF phospho-tau T181 and T217 biomarkers for Alzheimer's disease diagnosis. Alzheimer's & dementia : the journal of the Alzheimer's Association, 17(5), 755–767. <https://doi.org/10.1002/alz.12236>
46. Lam, S., Lipton, R. B., Harvey, D. J., Zammit, A. R., Ezzati, A., & Alzheimer's Disease Neuroimaging Initiative (2021). White matter hyperintensities and cognition across different Alzheimer's biomarker profiles. Journal of the American Geriatrics Society, 69(7), 1906–1915. <https://doi.org/10.1111/jgs.17173>
47. Ebenau, J. L., Timmers, T., Wesselman, L., Verberk, I., Verfaillie, S., Slot, R., van Harten, A. C., Teunissen, C. E., Barkhof, F., van den Bosch, K. A., van Leeuwenstijn, M., Tomassen, J., Braber, A. D., Visser, P. J., Prins, N. D., Sikkes, S., Scheltens, P., van Berckel, B., & van der Flier, W. M. (2020). ATN classification and clinical progression in subjective cognitive decline: The SCIENCe project. Neurology, 95(1), e46–e58. <https://doi.org/10.1212/WNL.0000000000009724>
48. Contador, J., Pérez-Millán, A., Tort-Merino, A., Balasa, M., Falgàs, N., Olives, J., Castellví, M., Borrego-Écija, S., Bosch, B., Fernández-Villullas, G., Ramos-Campoy, O., Antonell, A., Bargalló, N., Sanchez-Valle, R., Sala-Llonch, R., Lladó, A., & Alzheimer's Disease Neuroimaging Initiative (2021). Longitudinal brain atrophy and CSF biomarkers in early-onset Alzheimer's disease. NeuroImage. Clinical, 32, 102804. <https://doi.org/10.1016/j.nicl.2021.102804>
49. Eckerström, C., Svensson, J., Kettunen, P., Jonsson, M., & Eckerström, M. (2021). Evaluation of the ATN model in a longitudinal memory clinic sample with different underlying disorders. Alzheimer's & dementia (Amsterdam, Netherlands), 13(1), e12031. <https://doi.org/10.1002/dad2.12031>
50. Bucci, M., Chiotis, K., Nordberg, A., & Alzheimer’s Disease Neuroimaging Initiative (2021). Alzheimer's disease profiled by fluid and imaging markers: tau PET best predicts cognitive decline. Molecular psychiatry, 26(10), 5888–5898. <https://doi.org/10.1038/s41380-021-01263-2>
51. Rosenberg, A., Solomon, A., Soininen, H., Visser, P. J., Blennow, K., Hartmann, T., Kivipelto, M., & LipiDiDiet clinical study group (2021). Research diagnostic criteria for Alzheimer's disease: findings from the LipiDiDiet randomized controlled trial. Alzheimer's research & therapy, 13(1), 64. <https://doi.org/10.1186/s13195-021-00799-3>
52. Mondragón, J. D., Maurits, N. M., De Deyn, P. P., & Alzheimer's Disease Neuroimaging Initiative (2021). Functional connectivity differences in Alzheimer's disease and amnestic mild cognitive impairment associated with AT(N) classification and anosognosia. Neurobiology of aging, 101, 22–39. <https://doi.org/10.1016/j.neurobiolaging.2020.12.021>
53. Ou, Y. N., Xu, W., Li, J. Q., Guo, Y., Cui, M., Chen, K. L., Huang, Y. Y., Dong, Q., Tan, L., Yu, J. T., & Alzheimer’s Disease Neuroimaging Initiative (2019). FDG-PET as an independent biomarker for Alzheimer's biological diagnosis: a longitudinal study. Alzheimer's research & therapy, 11(1), 57. <https://doi.org/10.1186/s13195-019-0512-1>
54. Kern, S., Zetterberg, H., Kern, J., Zettergren, A., Waern, M., Höglund, K., Andreasson, U., Wetterberg, H., Börjesson-Hanson, A., Blennow, K., & Skoog, I. (2018). Prevalence of preclinical Alzheimer disease: Comparison of current classification systems. Neurology, 90(19), e1682–e1691. <https://doi.org/10.1212/WNL.0000000000005476>
55. Ottoy, J., Niemantsverdriet, E., Verhaeghe, J., De Roeck, E., Struyfs, H., Somers, C., Wyffels, L., Ceyssens, S., Van Mossevelde, S., Van den Bossche, T., Van Broeckhoven, C., Ribbens, A., Bjerke, M., Stroobants, S., Engelborghs, S., & Staelens, S. (2019). Association of short-term cognitive decline and MCI-to-AD dementia conversion with CSF, MRI, amyloid- and 18F-FDG-PET imaging. NeuroImage. Clinical, 22, 101771. <https://doi.org/10.1016/j.nicl.2019.101771>
56. De Kort, A. M., Kuiperij, H. B., Kersten, I., Versleijen, A., Schreuder, F., Van Nostrand, W. E., Greenberg, S. M., Klijn, C., Claassen, J., & Verbeek, M. M. (2021). Normal cerebrospinal fluid concentrations of PDGFRβ in patients with cerebral amyloid angiopathy and Alzheimer's disease. Alzheimer's & dementia : the journal of the Alzheimer's Association, 10.1002/alz.12506. Advance online publication. <https://doi.org/10.1002/alz.12506>
57. Pillai, J. A., Khrestian, M., Bena, J., Leverenz, J. B., & Bekris, L. M. (2021). Temporal Ordering of Inflammatory Analytes sTNFR2 and sTREM2 in Relation to Alzheimer's Disease Biomarkers and Clinical Outcomes. Frontiers in aging neuroscience, 13, 676744. <https://doi.org/10.3389/fnagi.2021.676744>
58. Jang, J. W., Kim, Y., Kim, S., Park, S. W., Kwon, S. O., Park, Y. H., Lim, J. S., Youn, Y. C., Hun Kim, S., Kim, S., & Alzheimer's Disease Neuroimaging Initiative (2020). Dynamic association between AT(N) profile and cognition mediated by cortical thickness in Alzheimer's continuum. NeuroImage. Clinical, 27, 102282. <https://doi.org/10.1016/j.nicl.2020.102282>
59. Li, K., Wang, S., Luo, X., Zeng, Q., Jiaerken, Y., Xu, X., Wang, C., Liu, X., Li, Z., Zhao, S., Zhang, T., Fu, Y., Chen, Y., Liu, Z., Zhou, J., Huang, P., & Zhang, M. (2020). Progressive Memory Circuit Impairments along with Alzheimer's Disease Neuropathology Spread: Evidence from in vivo Neuroimaging. Cerebral cortex (New York, N.Y. : 1991), 30(11), 5863–5873. <https://doi.org/10.1093/cercor/bhaa162>
60. Li, K., Luo, X., Zeng, Q., Huang, P., Shen, Z., Xu, X., Xu, J., Wang, C., Zhou, J., Zhang, M., & Alzheimer's Disease Neuroimaging Initiative (2019). Gray matter structural covariance networks changes along the Alzheimer's disease continuum. NeuroImage. Clinical, 23, 101828. <https://doi.org/10.1016/j.nicl.2019.101828>
61. Tsantzali, I., Boufidou, F., Sideri, E., Mavromatos, A., Papaioannou, M. G., Foska, A., Tollos, I., Paraskevas, S. G., Bonakis, A., Voumvourakis, K. I., Tsivgoulis, G., Kapaki, E., & Paraskevas, G. P. (2021). From Cerebrospinal Fluid Neurochemistry to Clinical Diagnosis of Alzheimer's Disease in the Era of Anti-Amyloid Treatments. Report of Four Patients. Biomedicines, 9(10), 1376. <https://doi.org/10.3390/biomedicines9101376>
62. Zhu, Z., Zeng, Q., Kong, L., Luo, X., Li, K., Xu, X., Zhang, M., Huang, P., & Yang, Y. (2021). Altered Spontaneous Brain Activity in Subjects With Different Cognitive States of Biologically Defined Alzheimer's Disease: A Surface-Based Functional Brain Imaging Study. Frontiers in aging neuroscience, 13, 683783. <https://doi.org/10.3389/fnagi.2021.683783>
63. Bubu, O. M., Umasabor-Bubu, O. Q., Turner, A. D., Parekh, A., Mullins, A. E., Kam, K., Birckbichler, M. K., Mukhtar, F., Mbah, A. K., Williams, N. J., Rapoport, D. M., de Leon, M., Jean-Louis, G., Ayappa, I., Varga, A. W., Osorio, R. S., & and Alzheimer's Disease Neuroimaging Initiative (2020). Self-reported obstructive sleep apnea, amyloid and tau burden, and Alzheimer's disease time-dependent progression. Alzheimer's & dementia : the journal of the Alzheimer's Association, 10.1002/alz.12184. Advance online publication. <https://doi.org/10.1002/alz.12184>
64. Sato, K., Mano, T., Suzuki, K., Toda, T., Iwatsubo, T., Iwata, A., & for Alzheimer’s Disease Neuroimaging Initiative (2020). Attempt to Predict A/T/N-Based Alzheimer's Disease Cerebrospinal Fluid Biomarkers Using a Peripheral Blood DNA Methylation Clock. Journal of Alzheimer's disease reports, 4(1), 287–296. <https://doi.org/10.3233/ADR-200205>
65. Park, S. W., Kim, S., Park, J., Jang, J. W., & Kim, S. (2020). A Comprehensive Visual Rating Scale for Predicting Progression from Mild Cognitive Impairment to Dementia in Patients with Alzheimer's Pathology or Suspected Non-Alzheimer's Pathology. Dementia and neurocognitive disorders, 19(4), 129–139. <https://doi.org/10.12779/dnd.2020.19.4.129>
66. Mattsson, N., Cullen, N. C., Andreasson, U., Zetterberg, H., & Blennow, K. (2019). Association Between Longitudinal Plasma Neurofilament Light and Neurodegeneration in Patients With Alzheimer Disease. JAMA neurology, 76(7), 791–799. <https://doi.org/10.1001/jamaneurol.2019.0765>
67. Lin, R. R., Xue, Y. Y., Li, X. Y., Chen, Y. H., Tao, Q. Q., & Wu, Z. Y. (2021). Optimal Combinations of AT(N) Biomarkers to Determine Longitudinal Cognition in the Alzheimer's Disease. Frontiers in aging neuroscience, 13, 718959. <https://doi.org/10.3389/fnagi.2021.718959>
68. Williams, H. (2020). Statistics for Politics and International Relations Using IBM SPSS Statistics. Statistics for Politics and International Relations Using IBM SPSS Statistics, 1-408.
